# When Do Generics Feel Justifiable? A Registered Report Bridging Key Theories

**DOI:** 10.5334/joc.493

**Published:** 2026-03-05

**Authors:** Felix Hermans, Walter Schaeken, Susanne Bruckmüller, Vera Hoorens

**Affiliations:** 1 Faculty of Psychology and Educational Sciences, KU Leuven, Belgium; 2 Department of Psychology, Friedrich-Alexander-Universität, Erlangen-Nürnberg, Germany

**Keywords:** Categorisation, Semantics, Mathematical modeling, Social cognition, Sentence processing

## Abstract

Bare plural generics (‘generics’ for short) attribute a feature to members of a category without specifying how many actually possess the feature (e.g., ‘Belgians love fries’). Generics are often used to perpetuate stereotypes and misinformation, yet researchers from many different fields disagree about how people decide what justifies a generic. This has led to a wide variety of theories on how people reason with generics. Pragmatic theories state that people find generics justifiable if they express knowledge that is useful (e.g., for survival or efficient transmission of knowledge). Statistical theories state that people find generics justifiable if the distribution of the features in the involved categories satisfies certain criteria. We compared the predictions of several influential theories in a registered study where participants, in each trial, saw the distribution of a feature in two fictitious groups. Participants then judged the justifiability of a generic that attributes the feature to one of the two groups. We independently manipulated the dangerousness of the features (a factor of relevance in pragmatic theories), absolute prevalence in the target group, and relative prevalence in the target group (factors of relevance in statistical theories). All experimental manipulations were within-participants. The order of conditions and the combinations of stimulus materials were fully randomized. Because our design allowed to predict specific, divergent patterns of main and interaction effects from each theory, this registered study allowed us to examine their relative merit and to further elucidate the cognitive mechanisms underlying generic justifiability.

‘Mosquitos carry the West Nile virus.’ ‘Canadians are right-handed.’ The first claim sounds justifiable, the second does not. But why? Stated more generally, how do people evaluate ‘bare plural generics’, plural statements that attribute features to the members of a category without quantifying how many of them actually possess that feature?

Answering this question is important because bare plural generics find application in many contexts (we will call them ‘generics’ for short as we focus on bare plural generics only; about other generics, see Gelman & Brandone, 2010; Geurts, 1985; Lerner & Leslie, 2016). They often occur in child-directed speech (Brandone et al., 2015; Gelman et al., 2004; Gelman & Roberts, 2017), everyday conversations among adults (Leslie, 2007; Mannheim, 2021), and scientific communication (DeJesus et al., 2019; Peters et al., 2022). They thus play a role in the acquisition of categories and in the formation and transmission of stereotypes (e.g., Berio & Musholt, 2023; Beukeboom & Burgers, 2019; Bosse, 2022; Gülgöz & Gelman, 2015; Hammond & Cimpian, 2017; Leslie, 2008; Rhodes et al., 2012; Silva, 2020), as well as in the misrepresentation of scientific findings (DeJesus et al., 2024).

However, understanding how people judge generics is not straightforward because of the ostensibly fickle patterns in their justifiability judgments. Some generics feel justifiable despite a low prevalence of the feature in the category. ‘Mosquitos carry the West Nile virus’ is a case in point as only around 1% of mosquitos do so (striking property generics; Leslie, 2008; Prasada et al., 2013). Other generics feel unjustifiable despite a high prevalence. ‘Canadians are right-handed’ is an example, as about 80% of Canadians are (majority false generic generalizations; Khemlani et al., 2012; Prasada et al., 2013).

So far, several different explanations of these patterns have been suggested. Most of them can be grouped into either a pragmatic or a statistical perspective. However, previous empirical research has usually only tested hypotheses based on one, or occasionally a few, of these competing explanations. In the present work, we aim to empirically bridge several key theories of generic justifiability. To do this, we have designed a registered experiment that allows these theories to make specific and diverging predictions. The design of the registered experiment thus enables powerful and unequivocal tests of these diverging predictions and allows us to compare the relative merit of several key theories of generic justifiability. In this way, this registered report contributes to the literature on generics by bridging existing theories and by stimulating further experimental research. Before describing the registered experiment, we first briefly review some pragmatic and statistical theories. We also briefly touch upon theories that we might call integrative or hybrid because they incorporate elements of both (for classifications of theories on generics, see Lazaridou-Chatzigoga et al., 2015; Leslie & Lerner, 2022; Tessler & Goodman, 2019b).

## Pragmatic, Statistical, and Hybrid Theories

Pragmatic (or conceptual) theories propose that generics are qualitatively different from quantified sentences, which use quantifiers like ‘some’, ‘most’, ‘many’, or ‘all’, and that the rules that determine whether quantified sentences are considered justified or not do not generally apply to generics in that they cannot be easily captured by a simple formula (Lazaridou-Chatzigoga et al., 2015). Instead, how readily people consider generics justifiable or not justifiable critically depends on the content of the described feature and the nature of the described group, in addition to the context in which the sentence is used (Tessler & Goodman, 2019b). For instance, in some contexts, considering a particular generic as justifiable may benefit survival (e.g., sharks bite swimmers), well-being (e.g., adults need 7 hours of sleep) or simply the efficient transmission of category knowledge (e.g., cats meow).

The most well-known representative of this family of theories is the *striking property hypothesis* (Cimpian, Brandone, et al., 2010; Leslie, 2008; Prasada et al., 2013). It states that people more readily find generics justifiable if they involve consequential (vs. neutral) features because it is evolutionarily advantageous to readily generalize consequential features – beneficial ones, and particularly, harmful ones. Failing to approach a member of a beneficial category, and even more so, failing to avoid a member of a harmful category is more problematic than needlessly avoiding or fruitlessly approaching a member of a neutral category, making undergeneralization more problematic than overgeneralization (Leslie, 2008). For example, ‘carrying the West Nile virus’ contains a dangerous feature, as being infected with this virus may have fatal consequences. It is therefore wise to judge the sentence ‘Mosquitos carry the West Nile virus’ to be justifiable, and, as a consequence, to avoid mosquito bites, no matter how few mosquitoes may carry the virus in reality. In the interest of clarity, we will henceforth refer to theories of this type as ‘pragmatic’ theories because theorists who espouse them often stress that generics ‘serve powerful pragmatic purposes’ (Gelman, 2021, pp. 520) and that pragmatic reasoning is a necessary underlying mechanism to explain the meaning of generics (e.g., Mannheim, 2021; Moty & Rhodes, 2021).

Statistical theories share the view that people decide whether generics are justifiable on purely quantitative grounds. They also differ from pragmatic theories in a methodological sense, as they often introduce specific formulas that make numerical predictions of how people might understand the meaning of generics, which can then be compared to empirical data (e.g., Kochari et al., 2020). Many of these theories involve the assumption that people engage in probabilistic reasoning. For example, Cohen (1999) assumed that people can read generics either absolutely (which he considered the default) or relatively. Under the absolute reading, generics feel justifiable if more than 50% of the members of the category have the involved feature. Under a relative reading, generics feel justifiable if the target feature is more prevalent in the target category than in other categories. Some authors use the concept ‘distinctiveness’ to denote the extent to which a feature is more prevalent in a target category than in an alternative category (Cella et al., 2022; Cimpian, Gelman, et al., 2010; Leslie, 2008). Furthermore, there are different ways to formalize how people might compare the prevalence of a feature between groups. Regardless of how one labels or formalizes the relative reading of generics, it may in any case explain why ‘Canadians are right-handed’ feels unjustifiable. There is no reason to believe that Canadians are right-handed more often than non-Canadians.

One point of discussion between the statistical and the pragmatic theories is the extent of intuitive statistical abilities of humans. For example, sentences like ‘Mosquitos carry the West Nile Virus’ signal that people sometimes make very strong overgeneralizations when reasoning with generics. Additionally, it has been shown that people sometimes also struggle reasoning with confidence intervals (Juslin et al., 2007) and that the motivation to systematically apply statistical principles of reason may vary from situation to situation (Lindskog et al., 2013). However, researchers have also shown that people are surprisingly efficient at applying basic principles of covariation detection (Lejarraga & Hertwig, 2021; Schulze & Hertwig, 2021), and that people sometimes apply statistical principles spontaneously, even if they lack any formal statistical schooling (Crocker, 1981; Lindskog et al., 2013; Peterson & Beach, 1967). Furthermore, it has been shown that even babies, non-human primates, and some bird species seem to rely on basic probabilistic reasoning skills when learning and reasoning about the features of a population (Denison et al., 2013; Denison & Xu, 2014; Schulze & Hertwig, 2021; Xu & Garcia, 2008). This registered report thus also examines the extent of human probabilistic reasoning skills that come into play when reasoning about generic justifiability.

Finally, hybrid theories state that both statistical factors (such as absolute and relative prevalence) and pragmatic or conceptual factors (such as dangerousness) should impact the justifiability of a generic (e.g., Tessler & Goodman, 2019b; Van Rooij & Schulz, 2020). For instance, Van Rooij and Schulz (2020) incorporated the conceptual notion of *emotional impact* (strongly related to strikingness) with statistical notions based on the psychology of associative learning. We will return to these three key perspectives and some of their variants in more detail below to elucidate the diverging predictions that they make in the context of our registered experiment (see: ‘Registered Study: Hypotheses and Predictions’).

## The Present Research

Earlier empirical research on the determinants of generic justifiability typically had one of two limitations. We will address these limitations in the present research.

First, many studies have tested one specific model rather than pitting competing models against each other. Because the variables of interest were therefore often confounded with variables of relevance to alternative models, the results were often open to alternative interpretations. Take, for example, studies that examine judgments of generics like ‘Mosquitos carry the West Nile virus’ or ‘Sharks bite swimmers’. The observation that most people find them justifiable despite the low absolute prevalence of the involved feature (here: ‘carrying the West Nile virus’ among mosquitos, or ‘biting swimmers’ among sharks) is consistent with both the striking property hypothesis (because of the dangerousness of the involved feature) and several statistical models (because of the higher relative prevalence of the feature among the involved animals than among other animals). It is therefore not clear from these studies whether generic justifiability in these examples is driven by high relative prevalence, by high strikingness, or by both.

Second, the studies that did compare competing models typically pitted one pragmatic model against one statistical model. However, a great variety of statistical models exist, and the various statistical models entail greatly diverging predictions. Even if findings are more consistent with, say, the striking property hypothesis than with a *particular* statistical model, that does not compellingly argue for the superiority of the striking property hypothesis over statistical models *in general*.

We therefore conducted a registered experiment that was designed to allow the predictions made by several specific statistical models and the striking property hypothesis to be tested at the same time. To this end, we simultaneously examined the effects of absolute prevalence, relative prevalence, and the dangerousness of features on the perceived justifiability of generics. We did so by means of a paradigm that we developed based on several earlier experiments on generics (Cella et al., 2022; Cimpian, Brandone, et al., 2010; Cimpian, Gelman, et al., 2010; Kochari et al., 2020; Lazaridou-Chatzigoga et al., 2019; Tessler & Goodman, 2019a, 2019b).

Participants, in a series of trials, judged the justifiability of a generic that ascribed a feature to one of two groups (henceforth called the ‘target group’; the other group being called the ‘referent group’) after having seen numerical information about the distribution of the feature (a physical or behavioral characteristic) across the two groups. The groups were fictitious (groups of aliens) to avoid confounding with, and non-systematic variability due to, pre-existing stereotypes. We independently manipulated the dangerousness of the features, the absolute prevalence in the target group, and the relative prevalence in the target group as compared to the referent group. The manipulation of relative prevalence happened via the features’ absolute prevalence in the referent group. Of course, many more referent groups might come to mind in real life situations. We therefore agree with Kochari et al., (2020) that a next step in understanding how people understand generics lies in uncovering how exactly people determine which referent group to compare the target group to. However, for the present study, we chose to include a single referent group for two reasons. First, we wished to manipulate relative prevalence in an unequivocal and straightforward manner. Second, convergent evidence exists for people’s tendency to think dichotomously (Kramer et al., 2021; Lux et al., 2024a, b; Moty & Rhodes, 2021). Dichotomous or binary thinking implies that examining how the distribution of features in two groups affects judgements of generics about one of the groups remains meaningful even if the involved groups are truly a small subset of many different groups. We have received ethical clearance for this study from the Social and Societal Ethics Committee (SMEC) and General Data Protection Regulation (GDPR) of the KU Leuven under the reference number G-2023-7266-R2(AMD).

## Non-registered Study: Pilot Study

In order to select the stimulus materials necessary for the registered study, we conducted an extensive pilot study.

### Pilot Study: Methods

The registered study required 36 fictitious alien group names, 9 dangerous features, and 9 non-dangerous features. To select appropriate fictitious names and features we collected 54 names, 12 dangerous features, and 12 non-dangerous features from previous experiments on generics (e.g., Cella et al., 2022; Cimpian, Brandone, et al., 2010). A native speaker of both English and Dutch translated the originally English materials into Dutch. This pilot study then assessed: (1) which names sounded neutral and were not consistently associated with any existing kind or group, (2) which dangerous features sounded most dangerous, and (3) which non-dangerous features sounded least dangerous. This pilot study received ethical clearance from the Social and Societal Ethics Committee (SMEC) and General Data Protection Regulation (GDPR) of the KU Leuven under the reference number G-2023-6836.

The sample consisted of 373 Dutch-speaking bachelor students of psychology, who participated for course credit (46 men, 320 women, 1 participant did not identify with one of these two categories, 1 participant did not wish to respond; age: *M* = 18.27, *SD* = 1.58, range 17–34). After having given informed consent, participants filled out an online questionnaire. To avoid tiredness and boredom, we presented each participant with only half of the list with fictitious names. For each of the 27 names on their list, participants rated how friendly, sincere, confident, capable, and dangerous they thought members of a group with that name would be. They responded on a 5-point Likert scale from 1 (not [trait] at all) to 5 (very [trait]). Participants also indicated whether they associated the name with an existing kind or group. If they answered that they did, we asked them to provide the name of this kind or group (open response format). For each feature, participants also rated how friendly, sincere, confident, capable, and dangerous they thought members of a kind with that feature would be on a 5-point Likert scale from 1 (not [trait] at all) to 5 (very [trait]). We counterbalanced the order of the tasks such that half of the participants provided ratings for the names first, while the other half provided ratings for the features first. We also randomized the order in which the names and the features in these two tasks were presented to participants to avoid sequence effects. Finally, participants indicated how seriously they had filled in the survey on a scale from 1 (not seriously at all) to 4 (very seriously). Six participants were excluded from the analyses because they indicated that they did not take the survey seriously at all.

### Pilot Study: Results

From the original list of 24 features, we selected those 9 features with the highest dangerousness ratings and those 9 with the lowest dangerousness ratings. Next, an independent Dutch-speaking rater identified, per fictitious group name, which kind or group it was most consistently associated with. We removed 16 names that were frequently associated with a single kind or group in an absolute sense (i.e., >10% of participants who had seen the name had associated it with the same kind or group) and/or in a relative sense (i.e., >50% of all associations for this name were with that kind or group). We combined an absolute and relative decision criterion, because, regardless of the number of associations per name, we particularly wanted to make sure we removed names that where consistently associated with the same kind or group. To achieve a final list of 36 names we removed 1 more name that was perceived as very friendly and sincere, and 1 more name that was perceived as very unfriendly, insincere, and dangerous. In this way we constructed a set of stimulus materials that were extensively piloted and highly controlled for the purpose of the main experiment. We report the selected stimulus materials for the main experiment in the Supplemental Materials.

## Registered Study: Methods

### Participants

For the registered study, we expected to recruit between 150 and 300 Dutch-speaking bachelor students of psychology to participate for course credit. The proposed range for the sample size was based on the reported sample sizes by Bian and Cimpian (2021), which were ‘considerably larger than those of older studies on this topic (e.g., Cimpian, Brandone, et al., 2010) and on par with sample sizes in more recent work (e.g., Tessler & Goodman, 2019b)’ (Bian & Cimpian, 2021, pp. 1838–1839). Moreover, since the sample sizes of Bian and Cimpian (2021) were not based on an a priori power analysis, we conducted a maximally conservative power analysis for a repeated measures, within-factors, ANOVA F-test using G*Power (version 3.1.9.7; Faul et al., 2007). For this power analysis, we set the correlation among repeated measures to 0 (which leads to an overestimation of the desired sample size if |actual correlation| is >0) and the nonsphericity correction parameter to 1. We estimated *f* based on the effect size reported for dangerousness in Bian and Cimpian (2021, Study 3: \[\eta _{p}^{2}=.035\]). We set desired power to .90 and α to .05. For a design with 1 group and 2 measurements, this power analysis indicated a minimal desired sample size of 147.

Student participation in research is organized in our university such that all bachelor students who are enrolled in an introductory research methods course must be given the opportunity to participate. Given the number of students who typically enrol in this course, a sample size within this range was feasible. However, it was difficult to predict the precise time frame that would be necessary to collect a sample size within our desired range. Therefore, we devised a clear stopping rule for this study: to achieve a sample size within this range, we planned to run the study for 8 weeks. After 8 weeks, we planned to stop collecting data if the minimum sample size of 150 had been reached. However, if the minimum sample size of 150 had not been reached at this point, we planned to run the study for another 8 weeks. We planned to continue this approach until we reached a sample size within the desired range. Finally, after we completed all our analyses, we planned to run a sensitivity analysis on the achieved sample in G*Power (version 3.1.9.7; Faul et al., 2007).

### Procedure

Potential participants were invited to a lab experiment that was announced as being on how people summarize scientific information when they try to do this truthfully but briefly (e.g., on social media) with only words and thus without numbers. We programmed the experiment in the PsychoPy3 Experiment Coder (Peirce et al., 2019) in Dutch. After having gone through the informed consent procedure and actively having given consent, participants indicated their age and gender (female, male, X, or prefer not to answer). At the start of the experiment, participants read the following introduction:

‘For this study, we would like you to imagine a team of astronaut-scientists. This team discovers various groups of aliens during an exploration of the universe. To learn more about these aliens the team observes random samples of alien groups on every planet it visits. During these observations the team collects data on the presence of various different features. Each trial you will see the data that the team has collected on a specific planet. This data will be about the presence of one feature in two groups of aliens living on that particular planet. After a few seconds a sentence will appear below the data. We ask you to indicate to what extent you find this sentence a justifiable conclusion based on the data. The speed with which you respond is not important.’

Participants were allowed to read all the information at their own pace and were instructed to press the spacebar key when they were ready to continue. They then first went through a practice trial that ostensively merely served to familiarize themselves with the use of the response slider but that also doubled as an outcome-neutral data quality check (see ‘Registered Study: Planned Analyses’):

‘To introduce you to the response slider that you will use in this study we will now ask you a short practice question. You may respond to this question by clicking anywhere on the slider below (except in the exact middle of the slider).

Suppose that: A leads to B and B leads to C

Conclusion: A leads to C

How justifiable do you think this conclusion is?’

After participants responded, they were reminded that they would have to use the same slider to indicate how justifiable they found certain conclusions during the experiment. They then had an opportunity to ask any question that they might have had. Once the whole procedure was clear to them, they continued to the actual experiment. Each trial started with a brief introduction of a fictitious planet and two groups of novel aliens living on it:

‘On a planet called [e.g., X-14-C], the team members identified two groups of aliens, which they called [e.g., Toabos] and [e.g., Ackeps]. In random samples of both groups, they observed how many members of both groups [e.g., sleep under trees].’

**Figure 1:**
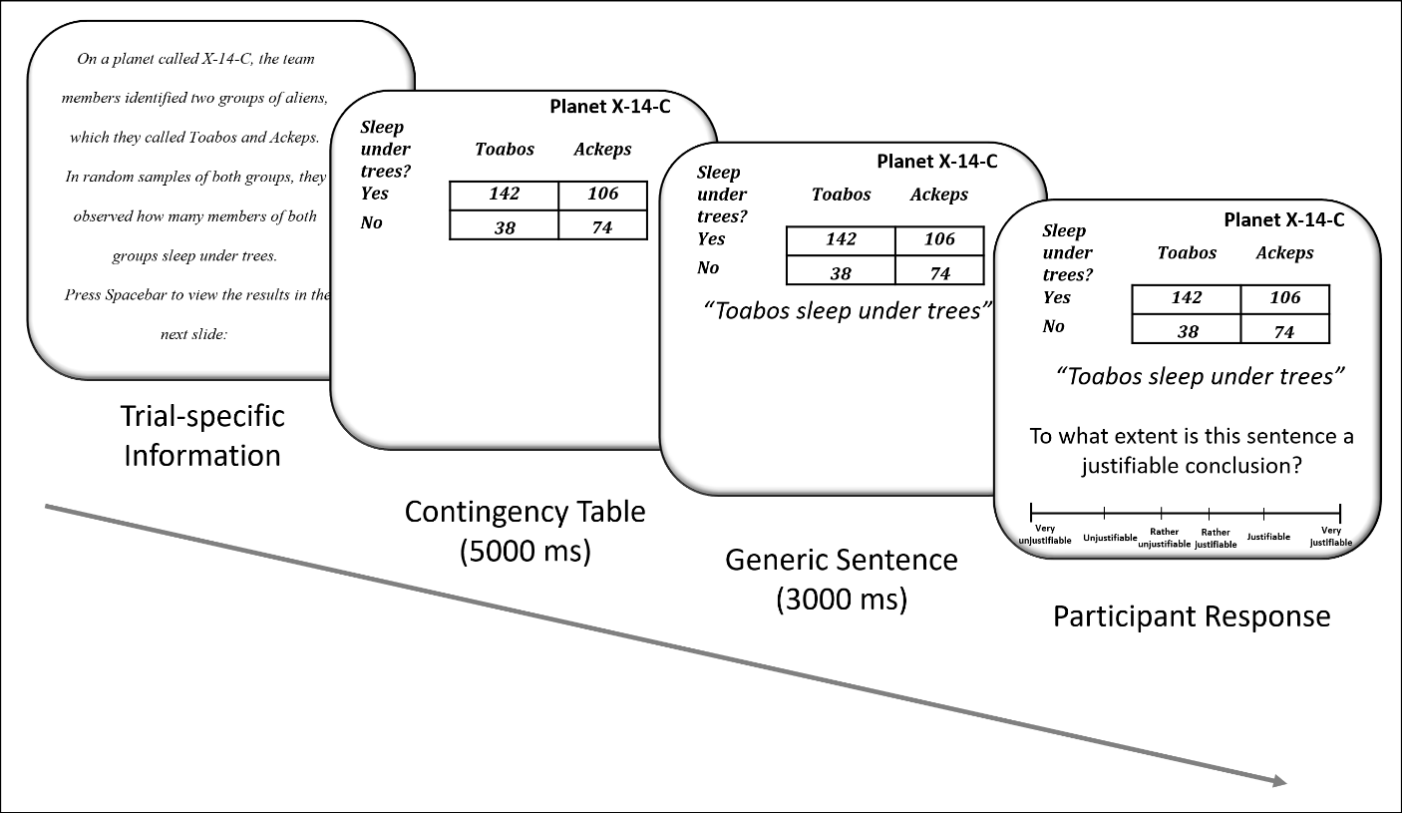
Schematic presentation of an experimental trial. Participants first saw trial-specific information about the involved planet, categories, and feature. The contingency table then appeared. After 5000 ms, the generic sentence appeared beneath it. Then, after an additional 3000 ms, the justifiability question also appeared and participants responded on a six point scale: (–3) very unjustifiable, (–2) unjustifiable, (–1) rather unjustifiable, (1) rather justifiable, (2) justifiable, (3) very justifiable.

Pressing the spacebar key made a contingency table appear with data about the distribution of the feature in the two alien groups (Figure 1). Participants were allowed to study this information at their own pace. After 5000 ms, a generic sentence appeared below the contingency table. The generic ascribed the feature to the group that on that trial functions as the target group (e.g., ‘Toabos sleep under trees’). After an additional 3000 ms, the question ‘To what extent is this sentence a justifiable conclusion?’ appeared, while the contingency table and the generic sentence remained visible. Participants answered on a continuous scale with six marked scale points, labelled as: (–3) very unjustifiable, (–2) unjustifiable, (–1) rather unjustifiable, (1) rather justifiable, (2) justifiable, (3) very justifiable. There was no marked neutral midpoint and participants were not be able to respond in the exact middle of the scale. Participants were thus forced to make a gradable, yet dichotomisable choice. Their answers formed the dependent variable.

After completing all trials, participants answered manipulation check questions for all three independent variables. First, we checked whether participants interpreted the levels of absolute and relative prevalence as intended. Participants saw a contingency table about the distribution of an unidentified feature (‘Feature X’) in two unidentified groups (Group H and Group K). The prevalence information for Group H came from a randomly drawn condition of absolute prevalence; the prevalence information for Group K came from a randomly drawn condition of relative prevalence. This contingency table remained on the screen for 5000 ms. After 5000 ms, the contingency table disappeared and participants answered two questions. As a manipulation check for the absolute prevalence manipulation, participants indicated approximately how many members of Group H had Feature X. They did so on a continuous scale with seven marked scale points, labelled as (–3) none, (–2) almost none, (–1) about a quarter, (0) about half, (1) about three quarters, (2) almost all, (3) all. As a manipulation check for the relative prevalence manipulation, participants indicated, on a categorical scale, whether Group H had Feature X (–1) less than Group K, (0) about as much as Group K, or (1) more than Group K.

Second, we checked whether participants perceived the features as intended. Participants again saw each feature in a random order. Participants indicated for each feature how dangerous they felt each feature was. They answered on a continuous scale with five marked scale points: (0) not at all dangerous, (1) a little bit dangerous, (2) rather dangerous, (3) dangerous, (4) very dangerous.

Next, participants responded to three final questions. First, we checked for the effect of a potential confounding variable that we used as a covariate. Previous research has shown that the effect of dangerousness can depend on whether the target group is perceived as human or non-human (Tasimi et al., 2017). We therefore had participants indicate to what extent the alien groups of this experiment were like humans on a continuous scale with six marked scale points, labelled: (–3) very unhuman, (–2) unhuman, (–1) somewhat unhuman, (1) somewhat human, (2) human, (3) very human. Second, we included a suspicion measure. Participants were prompted to express what they thought the research question was. Two condition-blind judges rated the open responses for references to (1) absolute prevalence, (2) relative prevalence, and (3) dangerousness. Third, we included a data quality check. Participants responded to the following question: ‘We would like to know how seriously you have participated. Having insight into that is important for a correct analysis of the results. Your answer does not influence how we register your participation. So please answer honestly.’ The response options were: ‘Not at all seriously’, ‘Not seriously’, ‘Not particularly seriously’, ‘Somewhat seriously’, ‘Seriously’, ‘Very seriously’. Earlier research has shown the validity of participants’ answer to such a question as an indicator of data quality (Arpinon & Espinosa, 2023; Aust et al., 2013; Verbree et al., 2020). Finally, we thanked participants and reminded them that they would receive an email with a detailed debriefing about the aims and design of the experiment after the data collection was finished.

### Design

The experiment had a fully within-participants design with 3(absolute prevalence: high, intermediate, low) × 3(relative prevalence: positive, equal, negative) × 2(dangerousness: dangerous, non-dangerous) conditions. The absolute prevalence conditions were: (1) high (about 75% of the target group members have the feature), (2) intermediate (about 50% of the target group members have the feature), (3) low (about 25% of the target group members have the feature). The relative prevalence conditions were: (1) positive (target group members have the feature about 20% more often than comparison group members), (2) equal (target group members have the feature about as often as comparison group members), (3) negative (target group members have the feature about 20% less often than comparison group members). To avoid that participants uncovered the different levels of absolute and relative prevalence, we programmed the experiment such that the sample size of each group was 180 on each trial (as compared to 100 – which would make it straightforward to uncover the underlying percentages), and such that the exact prevalence of the feature in both groups contained a small degree of randomness during each trial. The dangerousness conditions were: (1) dangerous, (2) non-dangerous. The order of the conditions was fully randomized to avoid confounding.

There was an exception to the aforementioned within-participants design. To keep the duration of the session reasonable and avoid undue repetitiveness, we manipulated the stimulus information in the manipulation check questions about absolute and relative prevalence between-participants. Participants were randomly assigned to one of the three absolute prevalence levels and one of the three relative prevalence levels.

### Stimulus Materials

In total, the full experiment required the following stimulus materials: 18 unique planet names, 36 fictitious alien group names, 9 dangerous features, and 9 non-dangerous features. To generate 18 unique planet names for each participant, the experiment for each trial randomly combined a letter, a double-digit number, and a second letter (e.g., planet A-01-C). To select appropriate fictitious names and features we conducted an extensive pilot study (see ‘Non-registered Study: Pilot Study’ and ‘Supplemental Materials’). For each participant, one unique planet name, two fictitious alien group names, and one dangerous or non-dangerous feature (depending on the dangerousness condition) was randomly selected for each trial. Each participant thus saw all the stimulus materials once, but always in a fully randomized order.

## Registered Study: Hypotheses and Predictions

### Pragmatic Theories: Focussing on the Striking Property Hypothesis

The striking property hypothesis predicts that people will find generics about dangerous features more easily justifiable than generics that involve equally prevalent non-dangerous features. Thus, the hypothesis predicts a main effect of dangerousness. Implicitly, it also predicts a main effect of absolute prevalence, because generics about the same feature (regardless of the dangerousness of the feature) should also become more justifiable as absolute prevalence increases. Furthermore, previous empirical studies showed greater effects of dangerousness at lower (vs. higher) levels of absolute prevalence (Bian & Cimpian, 2021). That is understandable, as there is more leeway for dangerousness to enhance the tendency to generalize from individual members to a category if the absolute prevalence of the feature in the category is low. Thus, an interaction of dangerousness with absolute prevalence would also be consistent with this perspective.

### Statistical Theories

Statistical theories hypothesize that the perceived justifiability of generics depends on the absolute prevalence of the feature in the target category and/or the relative prevalence of the feature in the target category as compared to the referent category. However, they differ in terms of the main and/or interaction effects that they predict. This is partly because they ascribe different weights to absolute vs. relative prevalence, and partly because they formalize relative prevalence differently. We here outline simulated predictions made by several statistical theories. Because these predictions are about our specific study, we formulate them in terms of features of members of ‘groups’ rather than of ‘categories’.

#### Absolute Prevalence

**Figure 2:**
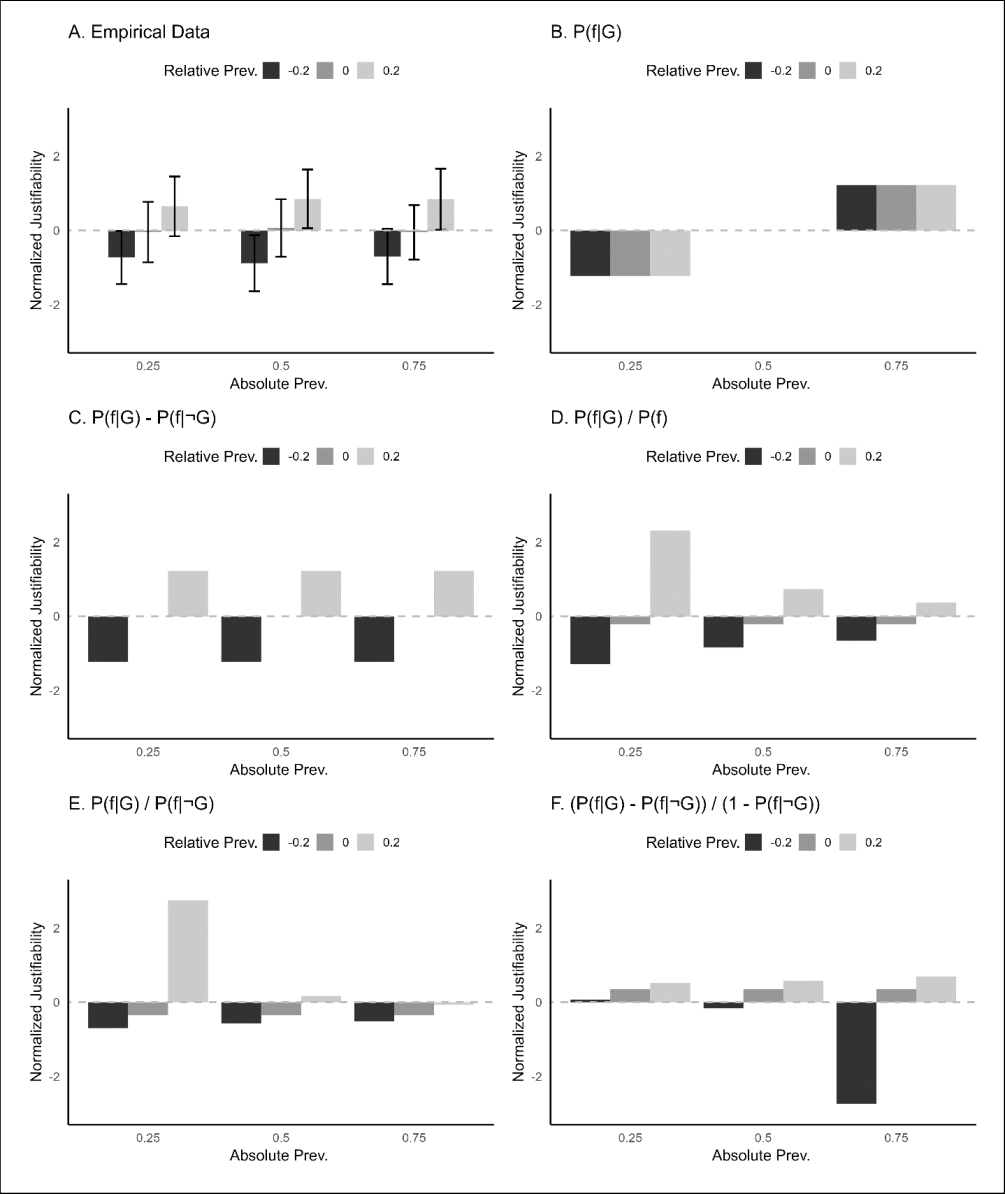
Histograms displaying predicted data patterns for the simulated empirical data and for each model. **A)** Normalized justifiability ratings provided by simulated participants, if participants rate justifiability using *P*(*f*|*G*)–*P*(*f*|¬*G*). **B)** Normalized justifiability ratings provided by a model where justifiability is predicted by absolute prevalence. **C)** Normalized justifiability ratings provided by a model where justifiability is predicted by a subtraction. **D)** Normalized justifiability ratings provided by a model where justifiability is predicted by a general fraction. **E)** Normalized justifiability ratings provided by a model where justifiability is predicted by a specific fraction. **F)** Normalized justifiability ratings provided by a model where justifiability is predicted by the fraction used in the model of Van Rooij and Schulz (2020).

One view holds that generics become more justifiable as the absolute prevalence of the feature in the target group increases (cf. the absolute reading of Cohen, 1999). Expressed more formally, this implies that the perceived justifiability of a generic is proportional to P(feature f|target group G). This view solely predicts a main effect of absolute prevalence, with a higher absolute prevalence entailing higher perceived justifiability. Figure 2B shows the pattern of justifiability judgments predicted by this view for the 3 (absolute prevalence) × 3 (relative prevalence) cells of the prevalence manipulations in the present experiment. For this and the subsequent statistical theories, we do not present the predictions separately for dangerous and non-dangerous features because they are identical.

#### Relative Prevalence: Difference Between Target and Referent

One view that ascribes a central role to relative prevalence states that people find generics more or less justifiable as a function of the difference between the absolute prevalence in the target group and the absolute prevalence in a referent group, with the referent group including all non-target groups combined (Kochari et al., 2020). This view can be formalized as a difference of probabilities: P(feature f|target group G)-P(feature f|referent group G’). This view solely predicts a main effect of relative prevalence, where an increase in relative prevalence entails an increase in perceived justifiability. Figure 2C shows the pattern of justifiability judgments predicted by this view.

#### Relative Prevalence: Ratio Prevalence in Target/Prior Prevalence

A related yet distinct statistical view with a central role for relative prevalence states that people find generics more or less justifiable as a function of the ratio between the absolute prevalence of the feature in the target group and the general prevalence of the feature (i.e., in all possible groups combined, including the target group). This view can be formalized as a fraction of probabilities: P(feature f|target group G)/P(feature f). This view predicts an interaction effect of absolute and relative prevalence, where the effect of relative prevalence on perceived justifiability increases when absolute prevalence decreases. In addition, this view also predicts a negative main effect of absolute prevalence (where an increase in absolute prevalence entails a decrease in perceived justifiability) and a positive main effect of relative prevalence (where an increase in relative prevalence entails an increase in perceived justifiability). Figure 2D shows the pattern of justifiability judgments predicted by this view. This figure shows that the predicted negative main effect of absolute prevalence for this experiment is mainly due to the strong positive perceived justifiability that is predicted in the condition that combines a low absolute prevalence with a positive relative prevalence.

#### Relative Prevalence: Ratio Prevalence in Target/Prevalence in Referent

Yet another statistical view with a central role for relative prevalence states that people compare the prevalence of a feature in a target group not with its prevalence in all groups combined or in all non-target groups combined, but with its prevalence in a specific alternative group that happens to be cognitively available – either because it is chronically available or because some situational factor makes it salient. This view rests upon evidence that human judgment and perception are inherently comparative (Lockhead, 2004; Manis et al., 1991; Mussweiler, 2003a, b), including when it comes to thinking about categories such as social groups (Bordalo et al., 2016; McCauley & Stitt, 1978; McCauley et al., 1980), and that whenever a referent category readily comes to mind when people consider a target category, people tend to engage in dichotomous thinking (Kramer et al., 2021). Any generic about a target category may thus provoke thoughts about how the target category differs from the referent category (Fraker, 2023).

This dichotomous statistical view is also formalized as a fraction of probabilities: P(feature f|target group G)/P(feature f|referent group G’). This view again predicts an interaction effect of absolute and relative prevalence, where the effect of relative prevalence on perceived justifiability increases when absolute prevalence decreases. In addition, this view also predicts a negative main effect of absolute prevalence (where an increase in absolute prevalence entails a decrease in perceived justifiability) and a positive main effect of relative prevalence (where an increase in relative prevalence entails an increase in perceived justifiability). Figure 2E shows the pattern of justifiability judgments predicted by this view. It is important to note that the pattern predicted by this theory merely differs from the pattern predicted by the former one in that the current theory predicts a stronger interaction between absolute and relative prevalence (this can be observed by comparing Figure 2D and Figure 2E).

### Hybrid Theories

Hybrid theories state that the perceived justifiability of generics jointly depends on the absolute and/or relative prevalence of the feature in the target category and on pragmatically relevant factors such as the dangerousness of the feature. We here take the hybrid theory of Van Rooij and Schulz (2020) as a case in point. According to this hybrid theory, justifiability should be formalized by multiplying a statistical term, \[{{\Delta }^{{\rm *}}}{\rm P}_{{\rm G}}^{{\rm f}}\], with a pragmatically relevant term, Value (f). In this hybrid theory, the statistical term is formalized as \[{{\Delta }^{*}}{\rm P}_{{\rm G}}^{{\rm f}}= \left[{\rm P}\left({\rm feature\ f} |{\rm target} ~{\rm group} ~{\rm G}\right)-{\rm P}\left({\rm feature\ f} |{\rm referent\ group\ G}\right)\right] 1-{\rm P}\left({\rm feature\ f} |{\rm referent\ group} ~{\rm G}\right)\]. The hybrid theory of Van Rooij and Schulz (2020) then multiplies this statistical term with Value(f), which represents the emotional impact of the feature in the generic. Notably, the inclusion of this term aligns with Leslie’s (2008) proposal that generics about striking, appalling, or gripping features are more easily accepted than generics discussing neutral features. This term is included because the emotional impact of events or features also matter when learning associations (Slovic et al., 2004, as cited by Van Rooij & Schulz, 2020). In this experiment, for example, Value (f) is equal to 1 for non-dangerous features, while for dangerous features Value(f) can be any value larger than 1 (Van Rooij & Schulz, 2020). Figure 2F shows the predictions that this hybrid theory makes for the non-dangerous properties in our experiment (because the hybrid theory reduces to the statistical term, since Value(f) = 1). For the dangerous features of our experiment, this hybrid theory does not specify how large Value(f) should be. Therefore, precisely predicting perceived justifiability for the dangerous features is not possible with this hybrid theory. Nonetheless, it predicts a similar pattern as for non-dangerous features in this case, be it with more extreme justifiability ratings (because the statistical term in the hybrid theory will now be multiplied by Value(f) > 1).

For our experiment, this hybrid theory predicts a two-way interaction between absolute prevalence and relative prevalence where the effects of relative prevalence on perceived justifiability become more pronounced when absolute prevalence increases (cf. Figure 2F). This interaction is thus markedly different from the interaction predicted by the previous two statistical theories, as the effect of relative prevalence on perceived justifiability is predicted by these previous theories to become more pronounced when absolute prevalence decreases. Depending on the magnitude of Value(f) for dangerous properties, a three-way interaction between absolute prevalence, relative prevalence, and dangerousness might also occur where the effects of relative prevalence on perceived justifiability become even more pronounced when features are also dangerous. In terms of main effects, this view predicts a positive main effect of relative prevalence where an increase in relative prevalence entails an increase in perceived justifiability. In addition, this view predicts negative main effects of absolute prevalence and of dangerousness for our experiment, where an increase in both entails a decrease in perceived justifiability. It is important to note that prediction of both negative main effects is mainly due to the strong negative perceived justifiability that is predicted in the condition that combines a high absolute prevalence with a negative relative prevalence (cf. Figure 2F).

## Registered Study: Planned Analyses

The code of our analysis pipeline can be found in https://osf.io/jm284/files/e4yq8. We first explored the characteristics of our sample by reporting the sample size and descriptive statistics for gender (number of participants who identify as women, men, X, or who prefer not to answer) and age (mean, standard deviation, range). We ran several outcome-neutral tests to check the data quality. First, we checked the distribution of responses to the practice trial. Since, logically, participants should consider the sentence in this trial relatively justifiable, we excluded participants who indicated that this sentence was, in general, unjustifiable: i.e., a response below the midpoint of the scale. Next, to ensure that there was a sufficiently large variability in participant responses, we excluded participants from all subsequent analyses who responded between (–3) very unjustifiable and (–2) unjustifiable on at least 12 of the 18 trials (thus showing a floor effect) or responded between (2) justifiable and (3) very justifiable on at least 12 of the 18 trials (thus showing a ceiling effect). We also excluded participants who provided justifiability ratings with an average response time 3 standard deviations above or below the average response time of all participants (as that might suggest that they either did not consider the stimuli attentively or tried to memorize or actively interpret the stimulus information to achieve maximal consistency across items). Finally, we checked the distribution of participants’ responses to the seriousness question. We excluded those participants from all subsequent analyses who answered ‘Not at all seriously’. We reported how many participants were affected by each of these exclusion criteria. We only planned to conduct the analyses as described below if under 40% of the data reached these exclusion criteria (cf. Arpinon & Espinosa, 2023). In the unlikely case that more than 40% would have met the exclusion criteria, we planned to include ‘data quality’ as an independent variable for our analyses (thus grouping participants depending on whether or not they met the exclusion criteria: no = 0; yes = 1). If data quality significantly interacted with one or more of the independent variables, we planned to assess the cause of the poor data quality, revise the study’s protocol to address the issue, and reconduct the experiment.

To check whether our manipulation of the three independent variables was successful, we examined participants’ responses to the manipulation check questions. For the manipulation check of absolute prevalence, we checked whether the perceived absolute prevalence of the feature in Group H significantly varied with the absolute prevalence manipulation as intended (low < intermediate < high absolute prevalence). For the manipulation check of relative prevalence, we checked whether the perceived relative prevalence of the feature (i.e., its prevalence in Group H as compared to the prevalence in Group K) significantly varied with the relative prevalence manipulation as intended (negative < equal < positive relative prevalence, with the judged relative prevalence in the negative relative prevalence condition being below the scale midpoint, and the judged relative prevalence in the positive relative prevalence condition being above the scale midpoint). For the manipulation check of the dangerousness of the feature, we examined whether participants, as intended, judged the dangerous features to be significantly more dangerous than the non-dangerous features.

Next, we explored the data concerning the participants’ awareness of our specific manipulations and hypotheses, and thus identified participants who correctly guessed them. We first ran the following analyses with these participants included. Then, we reran the analyses with these participants excluded to assess whether foreknowledge and/or suspicion affected the results. Upon data exclusion, we again reported descriptive statistics for the demographic variables of the reduced sample.

To test the predictions, we submitted the justifiability ratings to a repeated measures 3(absolute prevalence) × 3(relative prevalence) × 2(dangerousness) ANOVA. We reported F-statistics, degrees of freedom, p-values, and effect sizes (partial eta squared) for all main effects and interactions. We also tested a version of this ANOVA where we included the extent to which participants perceive the aliens to be humanlike as a covariate. We also reported (Bonferroni corrected) paired-sample t-tests to check for significant differences between the different levels of our independent variables. This analysis allowed us to test the diverging predictions delineated above (see: ‘Registered Study: Hypotheses and Predictions’).

**Figure 3:**
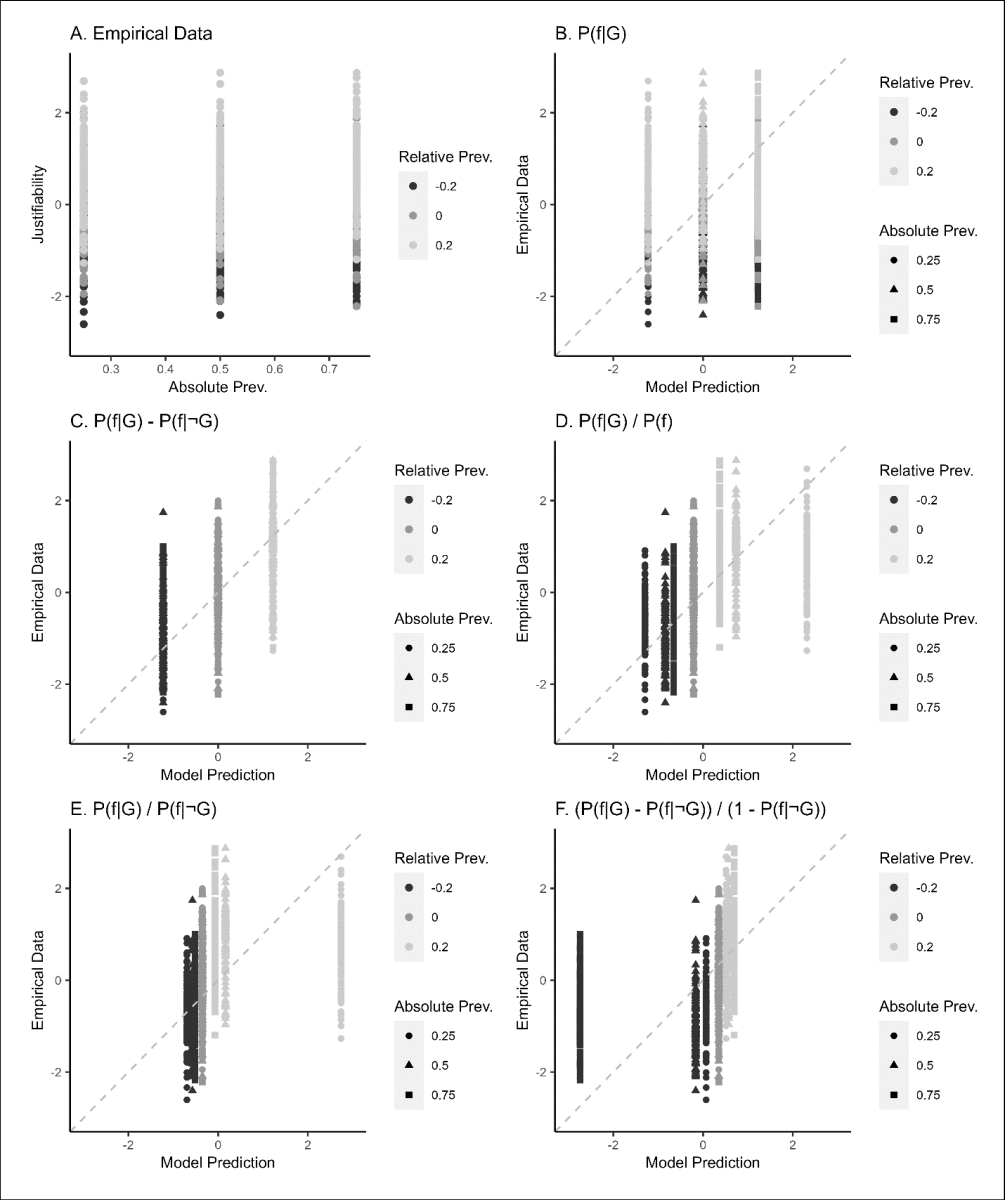
Scatterplots comparing simulated data to simulated predictions. **A)** justifiability rated by simulated participants when using *P*(*f*|*G*)–*P*(*f*|¬*G*). **B)** Fit of simulated data and justifiability predicted by absolute prevalence. **C)** Fit of the simulated data and justifiability predicted by a subtraction. **D)** Fit of the simulated data and justifiability predicted by a general fraction. **E)** Fit of the simulated data and justifiability predicted by a specific fraction. **F)** Fit of the simulated data and justifiability predicted by the fraction used in the model of Van Rooij and Schulz (2020).

To compare the pattern in the empirical data to the patterns predicted by the various hypotheses, we also planned to plot the empirical data like we have plotted the predicted patterns in Figure 2. Figure 2A is an example of such a graph (for illustrative purposes, we simulated data by adding noise to the predictions in Figure 2C). A visual comparison of this data plot to the various prediction plots would yield suggestive evidence concerning which models might be best suited to make general predictions of participant behaviour in this experiment. We also planned to report R-squared, Root Mean Square Error of Approximation (RMSEA),^1^ and Mean Absolute Error (MAE) fit statistics for each model, and a scatterplot comparing each prediction model to the empirical dataset (for an example, see Figure 3). We defined the best suited model as the model with the highest R-squared value, lowest RMSEA and MAE value, and a scatterplot showing the best match between the empirical data and the data as simulated on the basis of that model. We acknowledge that a good relative fit of a model is not necessarily a good indicator of model quality, especially if absolute model fit is low. Yet, since these models have all been based on theoretical considerations, we consider the theoretical value of comparing the effects of these considerations on model fit essential, regardless of whether absolute model fit is high or low. These analyses enabled us to examine the relative merit of the key theories explored here. In this way, the present work helped bridge key theories on the underlying mechanisms of human reasoning about generic justifiability.

## Transparency and Openness

We report how we determined our sample size, and all manipulations. If Stage 1 review was successful, we planned to report all data exclusions (if any), and all measures at Stage 2 submission. All data, analysis code, and research materials is available on the Open Science Framework (https://osf.io/jm284/). We conducted all analyses in R, Version 4.3.2 (R Core Team, 2022). We used the package ggplot, version 3.4.4 (Wickham, 2016) to make the graphs of this manuscript. In addition, we also used packages included in the tidyverse (Wickham et al., 2019), caret (Kuhn, 2008), effectsize (Ben-Shachlar et al., 2025), gridExtra (Auguie & Antonov, 2017), ggpubr (Kassambara, 2025a), irr (Gamer et al., 2019), Matrix (Bates et al., 2025), r-statix (Kassambara, 2025b), readxl (Wickham & Bryan, 2025), and readr (Wickham et al., 2025). The full pre-registered protocol is available on the Open science Framework (https://osf.io/pmxg3/).

## Results

### Pre-registered Stopping Rule and Exclusions

We followed the pre-registered stopping rule by first running the study for 8 weeks, which yielded a sample of 146 participants. As the minimum sample size of 150 had not been reached, we therefore ran the study for another 8 weeks, which yielded an additional 98 participants (a laboratory log of the data collection is available at https://osf.io/jm284/files/up2nb/). After the second wave of data collection, the total sample thus included 244 participants (*M*_age_ = 18.49, *SD*_age_ = 1.07, range: [17, 25]), of whom 36 self-identified as men, 207 as women, and 1 participant did not specify their gender.

We first checked the outcome-neutral data quality tests. Of the 69 participants (28.28% of the total sample) who failed at least one outcome-neutral test, 64 failed the first test (i.e., a response below the midpoint of the scale on the practice trial), none failed the second test (i.e., a floor or ceiling effect in responses to the experimental trials), 6 failed the third test (responding too fast or too slow), and 5 failed the fourth test (i.e., indicated that they participated ‘not at all seriously’). As fewer than 40% of the participants failed at least one outcome-neutral test, we did not include ‘data quality’ as an independent variable in the analyses but excluded the participants who failed at least one outcome-neutral test. The final sample thus consisted of 175 participants (*M*_age_ = 18.45, *SD*_age_ = 1.09, range: [17,25]) of whom 24 identified as men, 150 identified as women and 1 did not specify their gender.

### Pre-registered Manipulation Checks

For the manipulation check item on absolute prevalence, we first submitted participants’ estimates of the prevalence of Feature X in Group H to a one-way between-subjects ANOVA with three levels of absolute prevalence (low, intermediate, high). Participants indeed distinguished between the levels of absolute prevalence, *F*(2, 172) = 31.35, *p* < .001, \[\eta _{p}^{2}=.267\]. As shown by Bonferroni corrected Welch’s *t*-tests, participants in the high absolute prevalence condition (*n* = 58, *M* = 3.74, *SD* = 0.81) gave higher estimates than participants in the intermediate absolute prevalence condition (*n* = 59, *M* = 3.15, *SD* = 0.60), *t*(105) = 4.54, *p* < .001, *d* = 0.84, and than participants in the low absolute prevalence condition (*n* = 58, *M* = 2.55, *SD* = 0.99), *t*(109) = 7.12, *p* < .001, *d* = 1.32. The difference between the latter two conditions was also significant, *t*(93) = 3.96, *p* < .001, *d* = 0.74.

For the manipulation check item on relative prevalence, we first submitted participants’ perceptions of the relative prevalence of Feature X (i.e., its prevalence in Group H compared to in Group K) to a one-way between-subjects ANOVA with three conditions of relative prevalence (negative, equal, positive). Participants indeed distinguished between the conditions of relative prevalence, *F*(2, 172) = 7.29, *p* = .001, \[\eta _{p}^{2}=.078\]. As shown by Bonferroni corrected Welch’s *t*-tests, participants in the positive relative prevalence condition (*n* = 59, *M* = 0.31, *SD* = 0.88) gave higher relative prevalence estimates than participants in the equal relative prevalence condition (*n* = 55, *M* = –0.07, *SD* = 0.26), *t*(69) = 3.16, *p* = .007, *d* = 0.58, and participants in the negative relative prevalence condition (*n* = 61, *M* = –0.20, *SD* = 0.89), *t*(118) = 3.11, *p* = .007, *d* = 0.57. The difference between the equal relative prevalence condition and the negative relative prevalence condition was not significant, *t*(71.3) = 1.04, *p* = .909, *d* = 0.19.

For the manipulation check items of dangerousness, we examined whether participants perceived the dangerous features to be more dangerous than the non-dangerous features. As expected, participants found dangerous features (*M* = 3.41, *SD* = 0.37) significantly more dangerous than non-dangerous features (*M* = 0.67, *SD* = 0.43), paired samples *t*-test: *t*(174) = –70.95, *p* < .001, *d* = –5.36.

### Pre-registered Suspicion Check

Two independent judges rated participants’ open responses to the suspicion check items. According to the interpretations proposed by Landis and Koch (1977), the inter-rater reliability was fair for references to absolute prevalence (Cohen’s κ = .22), substantial for references to relative prevalence (Cohen’s κ = .76), and moderate for references to dangerousness (Cohen’s κ = .54). A third independent judge resolved disagreements between the first two judges.

We identified 28 participants who correctly guessed at least one manipulation. We ran the analyses once with these participants included and once excluding them (yielding a sample of 147 participants, *M*_age_ = 18.47, *SD*_age_ = 1.14, range: [17, 25], 20 self-identified as men and 127 as women). The results for the reduced sample (see Supplemental Materials) were highly similar to the results presented below, the only difference being that a significant interaction of absolute prevalence and dangerousness that occurred in the pre-registered hypothesis tests was no longer significant in the reduced sample (*p* = .052, with \[\eta _{p}^{2}=.021\]). Because the effect sizes were highly similar across sample compositions, the difference may be due to a difference in statistical power.

### Pre-registered Hypothesis Tests

We submitted participants’ justifiability ratings to a repeated measures 3(absolute prevalence) × 3(relative prevalence) × 2(dangerousness) ANOVA. The main effect of absolute prevalence was significant, *F*(1.73, 300.17) = 1057.16, *p* < .001, \[\eta _{p}^{2}=.859\]. Bonferroni-corrected paired-samples *t*-tests showed that participants gave higher justifiability ratings on high absolute prevalence trials (*M* = 1.40, *SD* = 0.83) than on intermediate absolute prevalence trials (*M* = –0.16, *SD* = 0.98), *t*(174) = 29.24, *p* < .001, *d* = 2.21, and low absolute prevalence trials (*M* = –1.31, *SD* = 0.98), *t*(174) = 38.73, *p* < .001, *d* = 2.93. Participants also gave higher justifiability ratings on intermediate absolute prevalence trials than on low absolute prevalence trials, *t*(174) = 21.90, *p* < .001, *d* = 1.66.

The main effect of relative prevalence was also significant, *F*(1.90, 329.74) = 22.54, *p* < .001, \[\eta _{p}^{2}=.115\]. Bonferroni-corrected paired-samples t-tests showed that participants gave higher justifiability ratings on positive relative prevalence trials (*M* = 0.11, *SD* = 1.45) than on equal relative prevalence trials (*M* = –0.03, *SD* = 1.45), *t*(174) = 3.65, *p* = .001, *d* = 0.28, and negative relative prevalence trials (*M* = –0.15, *SD* = 1.45), *t*(174) = 6.11, *p* < .001, d = 0.46. Participants also gave higher justifiability ratings on equal relative prevalence trials than on negative relative prevalence trials, *t*(174) = 3.42, *p* = .002, *d* = 0.26. Several statistical theories had also predicted an interaction of absolute and relative prevalence. However, this interaction was not significant, *F*(3.79, 658.82) = 1.42, *p* = .229, \[\eta _{p}^{2}=.008\].

The main effect of dangerousness was not significant, *F*(1, 174) = 3.25, *p* = .073, \[\eta _{p}^{2}=.018\], but the interaction of dangerousness and absolute prevalence was, *F*(1.78, 310.52) = 3.31, *p* = .043, \[\eta _{p}^{2}=.019\]. We used Bonferroni-corrected paired-samples *t*-tests to compare justifiability ratings for dangerous and non-dangerous features per absolute prevalence level. When absolute prevalence was high, we found no significant difference between dangerous features (*M* = 1.39, *SD* = 0.87) and non-dangerous features (*M* = 1.41, *SD* = 0.79), *t*(174) = –0.48, *p* = 1.00, *d* = –0.04. When absolute prevalence was low, we also found no significant difference between dangerous features (*M* = –1.30, *SD* = 1.01) and non-dangerous features (*M* = –1.33, *SD* = 0.96), *t*(174) = 0.53, *p* = 1.00, *d* = 0.04. In contrast, when absolute prevalence was intermediate justifiability ratings were significantly higher for dangerous features (*M* = –0.09, *SD* = 0.98) than for non-dangerous features (*M* = –0.23, SD = 0.97), *t*(174) = 2.75, *p* = .020, *d* = 0.21. No other main or interaction effects were significant, *Fs* < 3.25, *ps* > .073, and \[\eta _{p}^{2}s<.018\].

### Pre-registered Check of Humanness as a Covariate

Participants generally did not rate the aliens to be humanlike (*M* = –0.38, *SD* = 1.38). In order to control for how humanlike participants rated the aliens, we included this variable as a continuous between-subjects covariate in an analysis of covariance (ANCOVA) with the same 3(absolute prevalence) × 3(relative prevalence) × 2(dangerousness) repeated measures structure as the already described ANOVA. The results were nearly identical to those of the ANOVA, with significant main effects of absolute prevalence, *F*(1.72, 297.62) = 1059.33, *p* < .001, \[\eta _{p}^{2}=.860\], and relative prevalence, *F*(1.89, 327.68) = 22.43, *p* < .001, \[\eta _{p}^{2}=.115\], and an interaction of absolute prevalence and dangerousness, *F*(1.78, 310.52) = 3.31, *p* = .043, \[\eta _{p}^{2}=.019\]. Again, no other main or interaction effects were significant, *Fs* < 3.23, *ps* > .073, and \[\eta _{p}^{2}s<.018\] (see Supplemental Materials for full results of the analyses including humanness as a covariate).

### Pre-registered Model Comparisons

**Figure 4:**
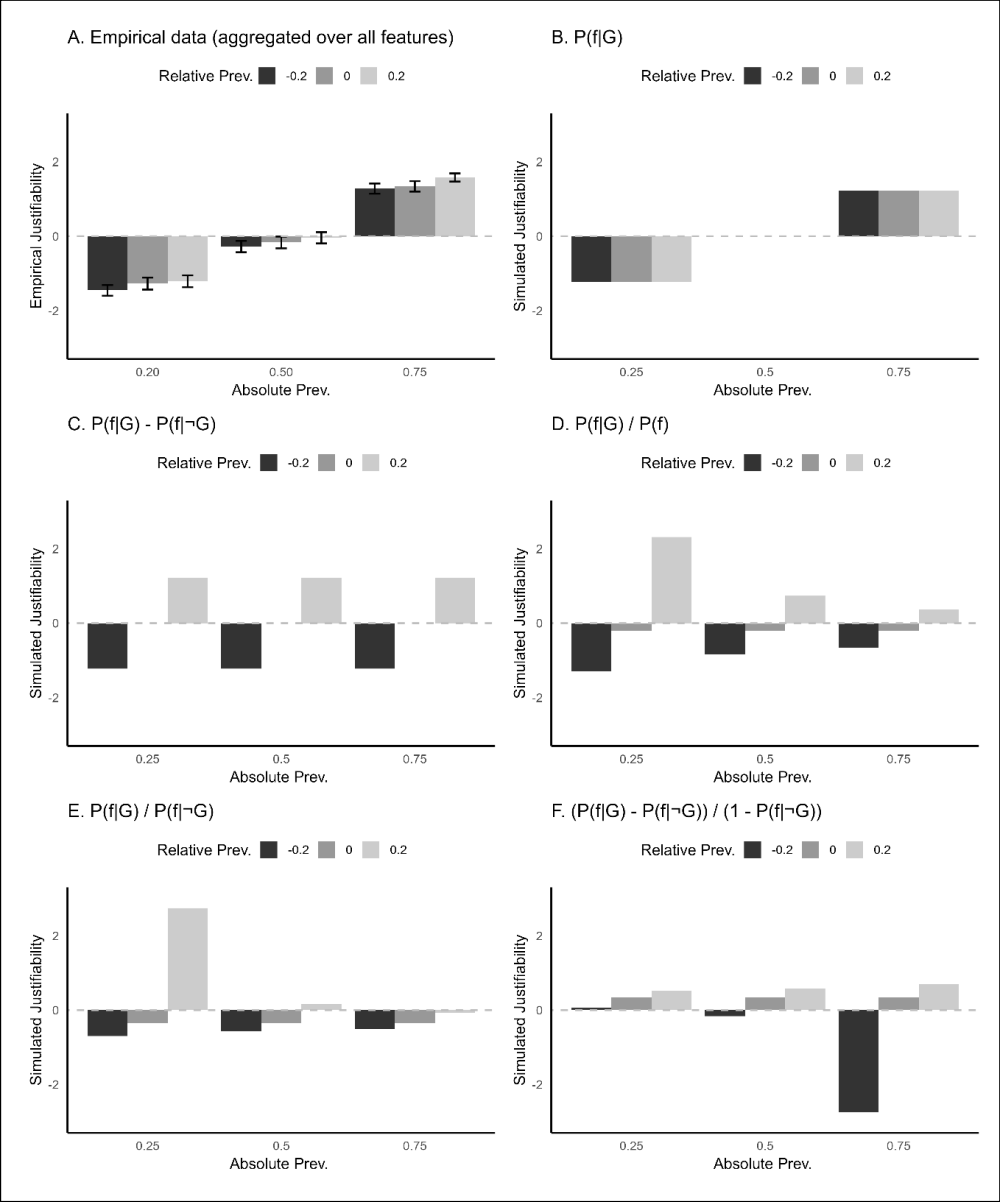
Histograms displaying the empirical data (*N* = 175) alongside the predicted data patterns for each model as was pre-registered (see Figure 2). **A)** Normalized empirical justifiability ratings provided by participants (error bars indicate 95% confidence intervals, corrected with the Cousineau-Morey method to take into account within-subject variability). **B)** Normalized justifiability ratings provided by a model where justifiability is predicted by absolute prevalence. **C)** Normalized justifiability ratings provided by a model where justifiability is predicted by a subtraction. **D)** Normalized justifiability ratings provided by a model where justifiability is predicted by a general fraction. **E)** Normalized justifiability ratings provided by a model where justifiability is predicted by a specific fraction. **F)** Normalized justifiability ratings provided by a model where justifiability is predicted by the fraction used in the model of Van Rooij and Schulz (2020).

**Figure 5:**
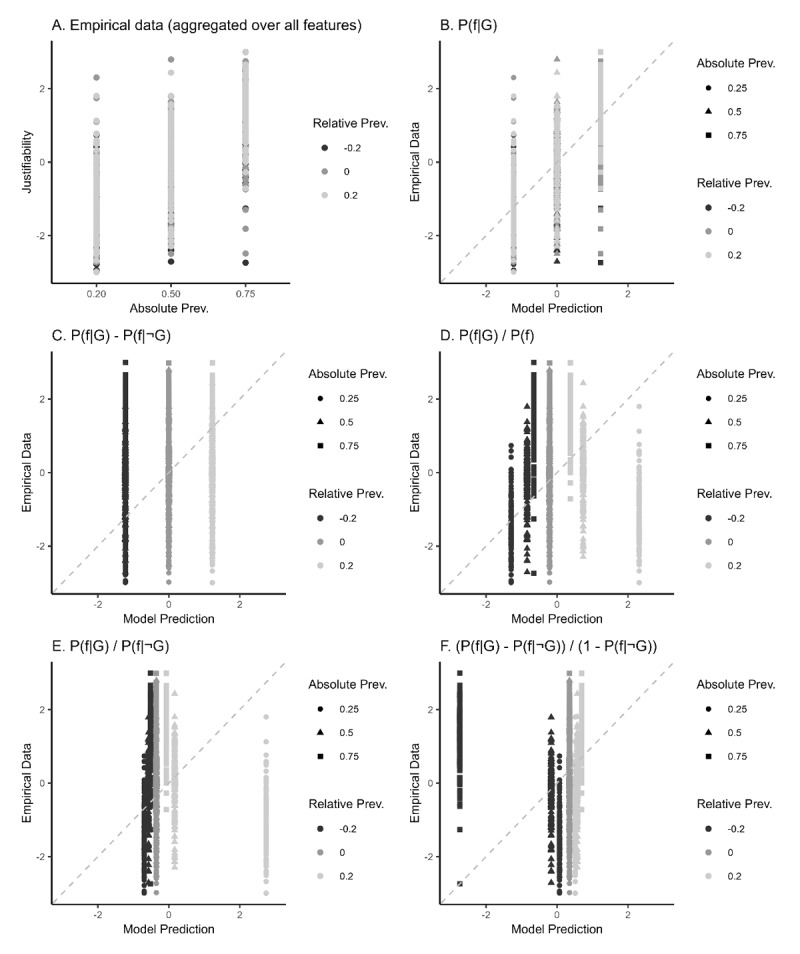
Scatterplots comparing empirical data (*N* = 175) to simulated predictions as pre-registered (see Figure 3). **A)** empirical justifiability ratings. **B)** Fit of simulated data and justifiability predicted by absolute prevalence. **C)** Fit of the simulated data and justifiability predicted by a subtraction. **D)** Fit of the simulated data and justifiability predicted by a general fraction. **E)** Fit of the simulated data and justifiability predicted by a specific fraction. **F)** Fit of the simulated data and justifiability predicted by the fraction used in the model of Van Rooij and Schulz (2020).

We compared the pattern in the empirical data to the patterns predicted by the various hypotheses presented above (Figure 4). The fit statistics confirmed the impression from a visual comparison of the empirical data and the various prediction patterns (see Figures 4 and 5) that the simple absolute prevalence model (see Figure 4B and 5B) captured the empirical data best. The absolute prevalence model accounted for 66.14% of the variance in the justifiability ratings of participants (*R^2^* = .6614, RMSE = 0.80, MAE = 0.62). The fit statistics favored this model over the subtraction model (Figure 4C and 5C; *R^2^* = .0061, RMSE = 1.63, MAE = 1.33), the general fraction model (Figure 4D and 5D; *R^2^* = .0060, RMSE = 1.75, MAE = 1.39), the specific fraction model (Figure 4E and 5E; *R^2^* = .0556, RMSE = 1.87, MAE = 1.46) and the fraction model of Van Rooij and Schulz (Figure 4F and 5F; *R^2^* = .0660, RMSE = 1.89, MAE = 1.47).

### Pre-registered Sensitivity Analysis

We conducted a post hoc sensitivity analysis in analogy to the a priori power analysis for this registered report, with *N* = 175. We were again maximally conservative and computed post hoc achieved power in a repeated measures, within-factors, ANOVA F-test using G*Power (version 3.1.9.7; Faul et al., 2007). Again, we set the correlation among repeated measures to 0, the nonsphericity correction parameter to 1, and α to .05. We estimated *f* based on the observed effect size for absolute prevalence (\[\eta _{p}^{2}~=.859\]), relative prevalence (\[\eta _{p}^{2}=.115\]), and dangerousness (\[\eta _{p}^{2}=.018\]).^2^ The post hoc sensitivity analysis yield a power of 1 for the main effects of absolute prevalence and relative prevalence (1 group and 3 measurements), and of .71 for the main effect of dangerousness (1 group and 2 measurements).

### Exploratory Analyses

Of all the participants excluded based on the pre-registered outcome-neutral data quality tests, the vast majority was excluded for having failed the first outcome-neutral data quality test (92.75%). Although we had expected that an attentive participant would rate the conclusion of the syllogism used in this test as justifiable, many participants must have hesitated to do so. In retrospect, some may have thought that the all-too-easy item was a trick question. If that was the case, failing the test would not necessarily mean that these participants were inattentive. In fact, the contrary may have been true.

We therefore ran explorative analyses from which we only excluded participants based on the other outcome-neutral tests. This entailed the exclusion of only 11 participants (4.51% of the total sample), leaving a sample of 233 participants (*M*_age_ = 18.48, *SD*_age_ = 1.06, range: [17, 25]) of whom 33 identified as men, 199 identified as women, and 1 did not specify their gender.

All manipulation checks were again successful. The results were highly similar to the results using the pre-registered exclusions (see Supplemental Materials). The main effect of dangerousness was now significant, *F*(1, 232) = 5.08, *p* = .025, \[\eta _{p}^{2}=.021\], but it was again completely driven by a dangerousness effect at the intermediate level of absolute prevalence, *t*(232) = –2.85, *p* = .014, *d* = –0.19. The dangerousness effect was not significant at the low and the high level of absolute prevalence (*ts* < .|–1.33|, *ps* > .555, and *ds* < |–.09|). Because the sample size was now larger than in the analyses presented above, we conducted another sensitivity analysis on the effect of dangerousness for this sample. We used an identical approach as above and estimated *f* based on the effect size we reported for dangerousness (\[\eta _{p}^{2}=.021\]) using the sample where participants were not removed based on the first outcome-neutral test (*N* = 233). This post hoc sensitivity analysis indicated a power of .88 for the effect of dangerousness in this sample.

## Discussion

Widely differing models exist of what makes people consider generics justifiable. We derived predictions from pragmatic, statistical, and hybrid perspectives and conducted a registered experiment to compare their relative merit. Participants saw contingency tables with information about the distribution of a feature in two fictitious alien groups, accompanied by a generic that ascribed the feature to one group, and judged how justifiable that generic was. Besides the dangerousness of the feature, we manipulated how prevalent the feature was in the group that it was ascribed to (absolute prevalence) and how much more or less prevalent it was in this group than in the alternative group (relative prevalence).

The absolute prevalence of the feature in the target group was the main determinant of participants’ ratings of how justifiable a generic that ascribed that feature to a target group was. However, the relative prevalence of the feature (i.e., its prevalence in the target group as compared to its prevalence in the alternative group) also played a role. The higher the absolute prevalence and the higher the relative prevalence of the feature, the more justifiable participants found the generic. In the present study, absolute prevalence was a much stronger determinant of the perceived justifiability of generics than relative prevalence was. However, it must be noted that the manipulation of relative prevalence was necessarily constrained. To enable us to create both negative and positive relative prevalence conditions at each level of absolute prevalence while prevalences had to remain within the 0–100% range, we limited relative prevalence to a maximum difference of 20% between the target group and a comparison group. Future work should explore stronger manipulations of relative prevalence by creating a more extreme contrast between the groups. This would be feasible, for instance, in experiments that only include conditions with intermediate to high absolute prevalence levels. This might also be feasible in experiments where the stimulus information involves much larger samples than the ones we used in the present experiment. Because the absolute number of category members can then still be quite high, even if the absolute prevalence is low, that would allow the creation of conditions where the features occur several times more frequently in the target category than in the alternative category. While the former approach would not allow for a (strong) manipulation of absolute prevalence and the latter approach might make the judgment tasks more complicated in the eyes of participants, both approaches should allow relative prevalence to have a stronger effect on generic justifiability (for a recently implemented approach, see Rothermund & Deutsch, 2025).

A more pragmatic factor, that is, the dangerousness of the feature, only played a role when the absolute prevalence level was intermediate. Participants rated generics about dangerous features as more justifiable than generics about non-dangerous features only when the features occurred in approximately 50% of the target group. This finding aligns with fuzzy logic approaches to semantics (e.g., Ramotowska et al., 2024; Zadeh, 1983). Fuzzy logic approaches typically find that participants are confident of their responses when numerical information is clear (e.g., very low or very high absolute prevalence). When numerical information is unclear (e.g., intermediate absolute prevalence) participants become less confident. They then consider other factors besides the numerical information. On the other hand, our finding of a dangerousness effect at an intermediate level of absolute prevalence seems to be at odds with results of earlier work (on the striking property hypothesis; Leslie, 2008) where the effect of dangerousness was strongest when absolute prevalence was low (e.g., Bian & Cimpian, 2021; Cimpian, Brandone, et al., 2010). Yet, an important difference between earlier research and ours is that we manipulated dangerousness and distinctiveness (operationalized as relative prevalence) independently. Moreover, we used a fully within-subjects design; each participant judged generics across all absolute and relative prevalence levels. Attentive participants may have therefore interpreted the information in the various conditions comparatively, which may have attenuated dangerousness effects at low prevalence levels. Future research should examine whether the interaction of absolute prevalence and dangerousness presented above replicates in designs where participants see only one level of absolute and relative prevalence.

Earlier work on pragmatic approaches to generics suggested that the striking property hypothesis only holds for generics about non-human groups (Tasimi et al., 2017). Our participants rated the alien groups as generally non-human and indeed gave higher justifiability ratings to generics describing dangerous versus non-dangerous features (albeit only at intermediate prevalence levels). Yet, including humanness judgments as a covariate did not alter the results. Although this could be taken to challenge the domain-specificity of the striking property hypothesis, it may reflect limited variability in the perceived humanness of the alien groups in the present study. Moreover, humanness was not a main focus of the present study. It was therefore measured rather than manipulated, precluding any causal interpretation. Future work could address this issue by extending the paradigm to include conditions with information and generics about real human groups.

### Bridging Theories of Generics and Directions for Integrative Modelling

As outlined in our predictions, a pragmatic theory (e.g., Leslie, 2008; Prasada et al., 2013) predicted that participants would consider generics about dangerous features more justifiable than generics about non-dangerous features. It also implied a positive main effect of absolute prevalence in combination with an interaction of absolute prevalence and dangerousness, with the effect of dangerousness being stronger at lower absolute prevalence levels (e.g., Bian & Cimpian, 2021). In our study, the effect of dangerousness was much smaller than the effects of absolute and relative prevalence, and dangerousness played a role at intermediate absolute prevalence levels, rather than at a low (or high) absolute prevalence level.

Statistical theories predicted that people would rate the justifiability of generics on the basis of only statistical information. In our study, participants indeed mainly relied on absolute prevalence and, to a lesser extent, on relative prevalence. Yet, they also took a pragmatic factor into account (the dangerousness of the feature). Of the statistical models that we tested, the one that only included absolute prevalence as a predictor fitted the data best (cf. the absolute reading of Cohen, 1999). Yet, this model does not account for the fact that at each level of absolute prevalence an increase in the relative prevalence of the feature in the target group also increased the perceived justifiability of a generic that ascribed the feature to that group. In addition, this model also does not account for the fact that increased dangerousness increased the perceived justifiability of generics at intermediate absolute prevalence levels.

Thus, while statistical and pragmatic accounts of generics have sometimes been considered unreconcilable (e.g., Carlson, 1995), our results underscore the importance of both statistical (absolute prevalence and relative prevalence) and pragmatic (dangerousness) factors as determinants of the perceived justifiability of generics. Rather than favoring either a pragmatic or a statistical perspective, we favor an integration of both into a hybrid theory. We already tested one hybrid theory here (Van Rooij & Schulz, 2020), yet this fraction model failed to capture the observed data. Still, other authors have increasingly advocated for hybrid approaches that integrate perspectives (e.g., Tessler & Goodman, 2019a, 2019b; Cohen, 2022; Lazaridou-Chatzigoga et al., 2015).

Most recently, a hybrid Contextual-Statistical, or ConStat, approach to the perceived validity of generics has been proposed (Hoorens et al., 2026). ConStat proposes that people evaluate descriptively interpreted generics^3^ as intuitive statisticians who compare absolute and relative statistical evidence against a flexible truth threshold which can vary depending on different pragmatic and contextual factors. This framework was explicitly described as being in ‘need for further specification’ (Hoorens et al., 2026, p. 12), as it characterizes how people understand generics without committing to a precise formal model. Therefore, integrating the present pattern of results with ConStat represents a promising avenue for future work aimed at developing a more precise, quantitatively predictive hybrid account.

Another promising direction for future research and integrative modeling on generics concerns individual differences in how participants weigh different sources of information when they encounter a generic. While the present analyses focused on aggregate patterns, it is plausible that some individuals might predominantly rely on absolute prevalence, while others may be more sensitive to other factors such as relative prevalence and dangerousness. Such differences may reflect distinct reasoning strategies to interpret generics. Future empirical work could investigate these individual differences more directly, for example by examining whether they are stable across tasks or linked to cognitive, linguistic, or educational factors. From a modeling perspective, incorporating individual-level variability may also help refine hybrid accounts of generics, moving beyond population-level predictions toward more personalized models of generic reasoning (for a recent model of semantic representations that has done so, see Ramotowska et al., 2023, 2024).

### Strengths, Limitations, and Future Directions

In order to reduce the gap between competing theories of how people reason with generics, it is necessary to compare specific numerical predictions to empirical data from human participants (e.g., Cohen, 2022; Kochari et al., 2020; Tessler & Goodman, 2019a, 2019b; Van Rooij & Schulz, 2020). Whereas earlier research has often focused on one or two predictive models at most, the present study examined the relative merit of five different models within a single experiment. Conducting such model-based comparisons poses substantial methodological challenges (Kochari et al., 2020), particularly with respect to ensuring that experimental designs allow for meaningful discrimination between competing prediction patterns. A key strength of the present work is that the experiment was explicitly designed to allow a fair comparison of multiple theoretical accounts. Moreover, the pre-registered nature of this experiment helped reduce researcher degrees of freedom in model testing and comparison. By integrating a formal modelling approach with controlled experimental methods, this work answers recent calls for more multimethod, interdisciplinary research on generics and for closer integration of experimental evidence into theory (e.g., Hoorens et al., 2026; Lazaridou-Chatzigoga et al., 2015).

Reliable hypothesis testing requires sufficiently large and well-justified samples. While earlier research on generics has sometimes relied on post hoc sensitivity analyses to evaluate whether a given sample was adequately powered (e.g., Bian & Cimpian, 2021), relatively few studies have explicitly based their sample sizes on a priori power analyses. In the present work, we conducted both an a priori power analysis and post hoc sensitivity analyses to transparently justify our sample size and assess the power of the observed effects. This allowed for a more nuanced understanding of findings across different sample compositions. Moreover, both the pilot study and the registered experiment were conducted in Dutch in independent samples of first-year bachelor students of psychology in the Dutch-speaking part of Belgium. Our study thus also follows recent recommendations to extend experimental research on generics to languages other than English (e.g., Castroviejo et al., 2023). Yet, in addition to well-justified sample sizes, we encourage future work to examine whether our findings generalize across age groups, languages, and cultural contexts.

In the present study, we employed a continuous response scale for justifiability rather than a binary true/false judgment or continuous truth-value or acceptability ratings (cf. Brandone et al., 2015; Passanisi et al., 2021, Ryu et al., 2022). This choice was motivated by a substantial methodological literature demonstrating that continuous or graded response scales preserve more information, increase sensitivity, and avoid distortions associated with dichotomization (e.g., Cohen, 1983; Dawes, 2008; Simms et al., 2019). Moreover, we assessed perceived justifiability rather than, perceived truth or acceptability. We made that choice in light of earlier work that suggests that ‘truth’ or ‘acceptability’ judgments may be more ambiguous than they seem. For example, judgments of the extent to which a generic is ‘true’ or ‘acceptable’ are sometimes conflated with perceptions of how desirable they are (Hoorens et al., 2026; Lux et al., 2024a). We reasoned that asking justifiability ratings would yield less ambiguous findings than asking truth or acceptability ratings because it would prompt participants to consider whether the available evidence justified the generic rather than whether they subjectively agreed with or applauded a generic. However, because experimental and theoretical work on generics has so far focused mostly on (often binary) truth-value or acceptability judgments, the present measure may limit the direct comparability of our findings with earlier work. Future experimental research should therefore systematically investigate how different judgment tasks shape evaluations of generics. For example, a direct comparison of binary and continuous justifiability, truth-value, and acceptability judgments within the present paradigm would reveal how participants interpret these different measures. This would help future researchers to carefully choose which response scale is the most appropriate in light of their research question and hypothesis.

Of course, future work that builds upon the present paradigm could consider implementing several additional methodological refinements (for an extensive overview of other methodological suggestions for experimental research on generics, see Hoorens et al., 2026). First, the syllogism which served as the first outcome-neutral data quality check could be made more transparent by using less ambiguous logical statements (e.g., transitive inference tasks such as “If A > B and B > C, then A > C”, Acuna et al., 2002; Riley, 1976).

Second, while the manipulation checks for absolute and relative prevalence were both successful, the effect sizes were stronger for the absolute prevalence check than for the relative prevalence check. As we already discussed, that was probably mainly because our manipulation of absolute prevalence may have been unintendedly stronger than our manipulation of relative prevalence. However, it must also be noted that participants always responded to the relative prevalence check second. That may have made it more difficult for participants to respond correctly due to an increased memory load. Future researchers may therefore not only manipulate relative prevalence more strongly but consider counterbalancing the order of the absolute and relative prevalence questions in the manipulation checks to reduce potential memory load.

Finally, previous accounts have highlighted the importance of contextual factors (e.g., the communication goal of the speaker) for generics (e.g., Greenberg, 2007; Hoorens et al., 2026; Rudolph, 2024; Sterken, 2015). The present study minimized such influences through tightly controlled stimulus materials, allowing participants to rely only on limited information about hypothetical alien groups. Still, this alien scenario constituted a specific context which may have affected participants’ judgments of generics. For example, the fact that we found much smaller evidence for pragmatic than for statistical models in the present work may in part be caused by the hypothetical nature, and therefore lower pragmatic relevance, of the alien scenario. Future research should extend the present paradigm by systematically varying contextual information to assess the impact of contextual factors on generic justifiability. Moreover, a combination of controlled experimental approaches with recent work that applies language models to natural language data may offer a promising interdisciplinary avenue to further disentangle the context-sensitivity of generics (for a recent example, see Cilleruelo et al., 2025).

## Conclusion

There are many competing views on what justifies generics, ranging from purely statistical to purely pragmatic accounts. In this registered report, we conducted an experiment designed to compare pragmatic, statistical, and hybrid theories by testing their predictions within a single experiment. Across analyses, statistical factors emerged as the strongest determinants of perceived generic justifiability, with a pragmatic factor playing a more constrained role. These findings suggest that neither purely statistical nor purely pragmatic accounts can provide a complete explanation of what makes generics justifiable. Instead, we show the need to develop hybrid theories that account for empirical data patterns by integrating multiple sources of information. Beyond these theoretical implications, the present results highlight the value of pre-registered, model-based comparisons, carefully justified sample sizes, and design choices that allow for meaningful discrimination between competing predictions. Together, this work contributes to ongoing efforts to develop an integrative theory of generics, and provides concrete directions for future research aimed at testing and refining such a theory.

## Data Accessibility Statement

The pre-registered protocol can be found at https://osf.io/pmxg3/. Data, analysis code, and study materials are available at https://osf.io/jm284/.

## Additional File

The additional file for this article can be found as follows:

10.5334/joc.493.s1 Supplementary Materials. Supplemental file including the feature list in English (with Dutch translations), the alien name list, and an overview of the full results for both the pre-registered and explorative analyses.

## Footnotes

It is important to note that although we pre-registered to report RMSEA (Root Mean Square Error of Approximation), this analysis would have been methodologically unsound. RMSEA is a fit index used in structural equation modeling to assess how well a model reproduces the population covariance matrix per degree of freedom and could thus not be calculated for this study. Instead, we report RMSE (Root Mean Squared Error), which is appropriate as it quantifies the average deviation of predicted values from observed values.

We only conducted sensitivity analyses for the main effects of the three independent variables in this study, since G*Power has been shown to drastically overestimate power for interaction effects (Baranger et al., 2023).

ConStat distinguishes between descriptive and normative interpretations of generics. While normative interpretations are not relevant for the generics under investigation in the present work, an extensive discussion of this distinction can be found in Hoorens et al. (2026, p. 4).

## References

[1] Acuna, B. D., Sanes, J. N., & Donoghue, J. P. (2002). Cognitive mechanisms of transitive inference. Experimental Brain Research, 146(1), 1–10. 10.1007/s00221-002-1092-y 12192572

[2] Arpinon, T., & Espinosa, R. (2023). A practical guide to Registered Reports for economists. Journal of the Economic Science Association, 9(1), 90–122. 10.1007/s40881-022-00123-1

[3] Auguie, B., & Antonov, A. (2017). gridExtra: Miscellaneous Functions for “Grid” Graphics (Version 2.3). https://cran.r-project.org/web/packages/gridExtra/

[4] Aust, F., Diedenhofen, B., Ullrich, S., & Musch, J. (2013). Seriousness checks are useful to improve data validity in online research. Behavioural Research, 45, 527–535. 10.3758/s13428-012-0265-2 23055170

[5] Baranger, D. A. A., Finsaas, M. C., Goldstein, B. L., Vize, C. E., Lynam, D. R., & Olino, T. M. (2023). Tutorial: Power Analyses for Interaction Effects in Cross-Sectional Regressions. Advances in Methods and Practices in Psychological Science, 6(3). 10.1177/25152459231187531 PMC12341451 40799847

[6] Bates, D., Maechler, M., Jagan, M., & Davis T. A. (2025). Matrix: Sparse and Dense Matrix Classes and Methods (Version 1.7–4). https://cran.r-project.org/web/packages/Matrix/index.html

[7] Ben-Shachar, M. S., Makowski, D., Lüdecke, D., Patil, I., Wiernik, B. M., Thériault, R., Kelley, K., Stanley, D., Caldwell, A., Burnett, J., Karreth, J., & Waggoner, P. (2025). effectsize: Indices of Effect Size (Version 1.0.1). https://cran.r-project.org/web/packages/effectsize/index.html

[8] Berio, L., & Musholt, K. (2023). How language shapes our minds: On the relationship between generics, stereotypes and social norms. Mind & Language, 38(4), 944–961. 10.1111/mila.12449

[9] Beukeboom, C. J., & Burgers, C. (2019). How stereotypes are shared through language: A review and introduction of the social categories and stereotypes communication (SCSC) framework. Review of Communication Research, 7, 1–37. 10.12840/issn.2255-4165.017

[10] Bian, L., & Cimpian, A. (2021). Generics about categories and generics about individuals: Same phenomenon or different? Journal of Experimental Psychology: Learning, Memory, and Cognition, 47(11), 1836. 10.1037/xlm0001100 34843339

[11] Bordalo, P., Coffman, K., Gennaioli, N., & Shleifer, A. (2016). Stereotypes. The Quarterly Journal of Economics, 131(4), 1753–1794. 10.1093/qje/qjw029

[12] Bosse, A. (2022). Stereotyping and generics. Inquiry, 0(0), 1–17. 10.1080/0020174X.2022.2074879

[13] Brandone, A. C., Gelman, S. A., & Hedglen, J. (2015). Children’s Developing Intuitions About the Truth Conditions and Implications of Novel Generics Versus Quantified Statements. Cognitive Science, 39(4), 711–738. 10.1111/cogs.12176 25297340 PMC4391975

[14] Carlson, G. N. (1995). Truth conditions of generic sentences: Two contrasting views. In G. N. Carlson & F. J. Pelletier (Eds.), The generic book (pp. 224–237). University of Chicago Press.

[15] Castroviejo, E., Hernández-Conde, J. V., Lazaridou-Chatzigoga, D., Ponciano, M., & Vicente, A. (2023). Are Generics Defaults? A Study on the Interpretation of Generics and Universals in 3 Age-Groups of Spanish-Speaking Individuals. Language Learning and Development, 19(3), 275–302. 10.1080/15475441.2022.2071715

[16] Cella, F., Marchak, K. A., Bianchi, C., & Gelman, S. A. (2022). Generic Language for Social and Animal Kinds: An Examination of the Asymmetry Between Acceptance and Inferences. Cognitive Science, 46(12), e13209. 10.1111/cogs.13209 36478284 PMC10078435

[17] Cilleruelo, G., Allaway, E., Haddow, B., & Birch, A. (2025). Generics are puzzling. Can language models find the missing piece? In O. Rambow, L. Wanner, M. Apidianaki, H. Al-Khalifa, B. D. Eugenio, & S. Schockaert (Eds.), Proceedings of the 31st International Conference on Computational Linguistics (pp. 6571–6588). Association for Computational Linguistics. https://aclanthology.org/2025.coling-main.438/

[18] Cimpian, A., Brandone, A. C., & Gelman, S. A. (2010). Generic Statements Require Little Evidence for Acceptance but Have Powerful Implications. Cognitive Science, 34(8), 1452–1482. 10.1111/j.1551-6709.2010.01126.x 21116475 PMC2992340

[19] Cimpian, A., Gelman, S. A., & Brandone, A. C. (2010). Theory-based considerations influence the interpretation of generic sentences. Language and Cognitive Processes, 25(2), 261–276. 10.1080/01690960903025227 20352078 PMC2843935

[20] Cohen, A. (1999). Think generic! The meaning and use of generic sentences. Doctoral dissertation, Carnegie Mellon University.

[21] Cohen, A. (2022). Genericity. In A. Cohen (Ed.), Oxford Research Encyclopedia of Linguistics. Oxford University Press. 10.1093/acrefore/9780199384655.013.326

[22] Cohen, J. (1983). The Cost of Dichotomization. Applied Psychological Measurement, 7(3), 249–253. 10.1177/014662168300700301

[23] Crocker, J. (1981). Judgment of covariation by social perceivers. Psychological Bulletin, 90(2), 272–292. 10.1037/0033-2909.90.2.272

[24] Dawes, J. (2008). Do Data Characteristics Change According to the Number of Scale Points Used? An Experiment Using 5-Point, 7-Point and 10-Point Scales. International Journal of Market Research, 50(1), 61–104. 10.1177/147078530805000106

[25] DeJesus, J. M., Callanan, M. A., Solis, G., & Gelman, S. A. (2019). Generic language in scientific communication. Proceedings of the National Academy of Sciences, 116(37), 18370–18377. 10.1073/pnas.1817706116 PMC6744883 31451665

[26] DeJesus, J. M., Callanan, M. A., Umscheid, V. A., & Gelman, S. A. (2024). Generic Language and Reporting Practices in Developmental Journals: Implications for Facilitating a More Representative Cognitive Developmental Science. Journal of Cognition and Development, 25(2), 273–295. 10.1080/15248372.2023.2290504

[27] Denison, S., Reed, C., & Xu, F. (2013). The emergence of probabilistic reasoning in very young infants: Evidence from 4.5- and 6-month-olds. Developmental Psychology, 49(2), 243–249. 10.1037/a0028278 22545837

[28] Denison, S., & Xu, F. (2014). The origins of probabilistic inference in human infants. Cognition, 130(3), 335–347. 10.1016/j.cognition.2013.12.001 24384147

[29] Faul, F., Erdfelder, E., Lang, A.-G., & Buchner, A. (2007). G*Power 3: A flexible statistical power analysis program for the social, behavioral, and biomedical sciences. Behavior Research Methods, 39(2), 175–191. 10.3758/bf03193146 17695343

[30] Fraker, W. (2023). Social kind generics and the dichotomizing perspective. Philosophical Psychology, 1–21. 10.1080/09515089.2023.2276307

[31] Gamer, M., Lemon, J., & Singh, I. F. P. (2019). irr: Various Coefficients of Interrater Reliability and Agreement (Version 0.84.1). https://cran.r-project.org/web/packages/irr/index.html

[32] Gelman, S. A. (2021). Generics in society. Language in Society, 50(4), 517–532. 10.1017/S0047404521000282

[33] Gelman, S. A., & Brandone, A. C. (2010). Fast-Mapping Placeholders: Using Words to Talk About Kinds. Language Learning and Development, 6(3), 223–240. 10.1080/15475441.2010.484413 22068229 PMC3007088

[34] Gelman, S. A., & Roberts, S. O. (2017). How language shapes the cultural inheritance of categories. Proceedings of the National Academy of Sciences, 114(30), 7900–7907. 10.1073/pnas.1621073114 PMC5544278 28739931

[35] Gelman, S. A., Taylor, M. G., Nguyen, S. P., Leaper, C., & Bigler, R. S. (2004). Mother-Child Conversations about Gender: Understanding the Acquisition of Essentialist Beliefs. Monographs of the Society for Research in Child Development, 69(1), i–142.

[36] Geurts, B. (1985). Generics. Journal of Semantics, 4(3), 247–255. 10.1093/jos/4.3.247

[37] Greenberg, Y. (2007). Exceptions to generics: Where vagueness, context dependence and modality interact. Journal of Semantics, 24(2), 131–167. 10.1093/jos/ffm002

[38] Gülgöz, S., & Gelman, S. A. (2015). Children’s recall of generic and specific labels regarding animals and people. Cognitive Development, 33, 84–98. 10.1016/j.cogdev.2014.05.002 25598575 PMC4292889

[39] Hammond, M. D., & Cimpian, A. (2017). Investigating the cognitive structure of stereotypes: Generic beliefs about groups predict social judgments better than statistical beliefs. Journal of Experimental Psychology: General, 146, 607–614. 10.1037/xge0000297 28459260

[40] Hoorens, V., Hermans, F., & Bruckmüller, S. (2026). Why boys cry and don’t cry: The Contextual-Statistical (ConStat) approach to the perceived validity of generics. Cognition, 266, 106323. 10.1016/j.cognition.2025.106323 40967131

[41] Juslin, P., Winman, A., & Hansson, P. (2007). The naïve intuitive statistician: A naïve sampling model of intuitive confidence intervals. Psychological Review, 114(3), 678–703. 10.1037/0033-295X.114.3.678 17638502

[42] Kassambara, A. (2025a). ggpubr: “ggplot2” Based Publication Ready Plots (Version 0.6.2). https://cran.r-project.org/web/packages/ggpubr/index.html

[43] Kassambara, A. (2025b). rstatix: Pipe-Friendly Framework for Basic Statistical Tests (Version 0.7.3). https://cran.r-project.org/web/packages/rstatix/index.html

[44] Khemlani, S., Leslie, S.-J., & Glucksberg, S. (2012). Inferences about members of kinds: The generics hypothesis. Language and Cognitive Processes, 27(6), 887–900. 10.1080/01690965.2011.601900

[45] Kochari, A., Van Rooij, R., & Schulz, K. (2020). Generics and Alternatives. Frontiers in Psychology, 11. 10.3389/fpsyg.2020.01274 PMC7347792 32719631

[46] Kramer, H. J., Goldfarb, D., Tashjian, S. M., & Hansen Lagattuta, K. (2021). Dichotomous thinking about social groups: Learning about one group can activate opposite beliefs about another group. Cognitive Psychology, 129, 101408. 10.1016/j.cogpsych.2021.101408 34330016

[47] Kuhn, M. (2008). Building Predictive Models in R Using the caret Package. Journal of Statistical Software, 28, 1–26. 10.18637/jss.v028.i0527774042

[48] Landis, J. R., & Koch, G. G. (1977). The Measurement of Observer Agreement for Categorical Data. Biometrics, 33(1), 159–174. 10.2307/2529310 843571

[49] Lazaridou-Chatzigoga, D., Katsos, N., & Stockall, L. (2015). Genericity is Easy? Formal and Experimental Perspectives. Ratio, 28(4), 470–494. 10.1111/rati.12116

[50] Lazaridou-Chatzigoga, D., Katsos, N., & Stockall, L. (2019). Generalizing About Striking Properties: Do Glippets Love to Play With Fire? Frontiers in Psychology, 10. 10.3389/fpsyg.2019.01971 PMC6727862 31555170

[51] Lejarraga, T., & Hertwig, R. (2021). How experimental methods shaped views on human competence and rationality. Psychological Bulletin, 147(6), 535–564. 10.1037/bul0000324 34843298

[52] Lerner, A., & Leslie, S.-J. (2016). Generics and Experimental Philosophy. In A Companion to Experimental Philosophy (pp. 404–416). John Wiley & Sons, Ltd. 10.1002/9781118661666.ch28

[53] Leslie, S.-J. (2007). Generics and the Structure of the Mind. Philosophical Perspectives, 21, 375–403. 10.1111/j.1520-8583.2007.00138.x

[54] Leslie, S.-J. (2008). Generics: Cognition and Acquisition. The Philosophical Review, 117(1), 1–47. 10.1215/00318108-2007-023

[55] Leslie, S.-J., & Lerner, A. (2022). Generic Generalizations. https://plato.stanford.edu/ENTRIES/generics/

[56] Lindskog, M., Winman, A., & Juslin, P. (2013). Calculate or wait: Is man an eager or a lazy intuitive statistician? Journal of Cognitive Psychology, 25(8), 994–1014. 10.1080/20445911.2013.841170

[57] Lockhead, G. R. (2004). Absolute judgments are relative: A reinterpretation of some psychophysical ideas. Review of General Psychology, 8(4), 265–272. 10.1037/1089-2680.8.4.265

[58] Lux, A., Bruckmüller, S., & Hoorens, V. (2024a). Are women brave or braver than Men? Judgments of implicit and explicit intergroup comparisons. Journal of Language and Social Psychology, 43(2), 137–163. 10.1177/0261927X231210513

[59] Lux, A., Bruckmüller, S., & Hoorens, V. (2024b). Interpretations of generic versus comparative statements about groups: Similarities and differences. Journal of Language and Social Psychology, online first. 10.1177/0261927X241292562

[60] Manis, M., Biernat, M., & Nelson, T. F. (1991). Comparison and expectancy processes in human judgment. Journal of Personality and Social Psychology, 61(2), 203–211. 10.1037/0022-3514.61.2.203 1920062

[61] Mannheim, B. (2021). The social (and cultural, and syntactic, and semantic) life of generics. Language in Society, 50(4), 605–618. 10.1017/S0047404521000336

[62] McCauley, C., & Stitt, C. L. (1978). An individual and quantitative measure of stereotypes. Journal of Personality and Social Psychology, 36(9), 929–940. 10.1037/0022-3514.36.9.929

[63] McCauley, C., Stitt, C. L., & Segal, M. (1980). Stereotyping: From prejudice to prediction. Psychological Bulletin, 87(1), 195–208. 10.1037/0033-2909.87.1.195

[64] Moty, K., & Rhodes, M. (2021). The unintended consequences of the things we say: What generic statements communicate to children about unmentioned categories. Psychological Science, 32(2), 189–203. 10.1177/0956797620953132 33450169 PMC8258311

[65] Mussweiler, T. (2003a). Comparison processes in social judgment: mechanisms and consequences. Psychological Review, 110(3), 472–489. 10.1037/0033-295X.110.3.472 12885111

[66] Mussweiler, T. (2003b). ‘Everything is relative’: Comparison processes in social judgment The 2002 Jaspars Lecture. European Journal of Social Psychology, 33(6), 719–733. 10.1002/ejsp.169

[67] Passanisi, A., Pace, U., Kabir, K. T., & Hampton, J. A. (2021). Ducks lay eggs and lions have manes: The acceptability of gender-specific minority generic sentences. Journal of Experimental Psychology: Learning, Memory, and Cognition, 47(12), 1998. 10.1037/xlm0001081 34807704

[68] Peirce, J., Gray, J. R., Simpson, S., MacAskill, M., Höchenberger, R., Sogo, H., Kastman, E., & Lindeløv, J. K. (2019). PsychoPy2: Experiments in behavior made easy. Behavior Research Methods, 51(1), 195–203. 10.3758/s13428-018-01193-y 30734206 PMC6420413

[69] Peters, U., Krauss, A., & Braganza, O. (2022). Generalization Bias in Science. Cognitive Science, 46(9), e13188. 10.1111/cogs.13188 36044007

[70] Peterson, C. R., & Beach, L. R. (1967). Man as an intuitive statistician. Psychological Bulletin, 68(1), 29–46. 10.1037/h0024722 6046307

[71] Prasada, S., Khemlani, S., Leslie, S.-J., & Glucksberg, S. (2013). Conceptual distinctions amongst generics. Cognition, 126(3), 405–422. 10.1016/j.cognition.2012.11.010 23291421

[72] R Core Team. (2022). R: A Language and Environment for Statistical Computing (Version 4.3.2). R Foundation for Statistical Computing. https://www.R-project.org/

[73] Ramotowska, S., Haaf, J., Van Maanen, L., & Szymanik, J. (2024). Most quantifiers have many meanings. Psychonomic Bulletin & Review, 31(6), 2692–2703. 10.3758/s13423-024-02502-7 38717681 PMC11680628

[74] Ramotowska, S., Steinert-Threlkeld, S., van Maanen, L., & Szymanik, J. (2023). Uncovering the Structure of Semantic Representations Using a Computational Model of Decision-Making. Cognitive Science, 47(1), e13234. 10.1111/cogs.13234 36640435

[75] Rhodes, M., Leslie, S.-J., & Tworek, C. M. (2012). Cultural transmission of social essentialism. Proceedings of the National Academy of Sciences, 109(34), 13526–13531. 10.1073/pnas.1208951109 PMC3427061 22869722

[76] Riley, C. A. (1976). The representation of comparative relations and the transitive inference task. Journal of Experimental Child Psychology, 22(1), 1–22. 10.1016/0022-0965(76)90085-0

[77] Rothermund, P., & Deutsch, R. (2025). Differentiation and Generic Sentences. Cognitive Science, 49(3), e70057. 10.1111/cogs.70057 40132075 PMC11936314

[78] Rudolph, R. E. (2024). Social Generics in Context. Philosophical Perspectives, 38(1), 103–116. 10.1111/phpe.12206

[79] Ryu, S. H., Yang, W., & Park, J. C. (2022). Flexible Acceptance Condition of Generics from a Probabilistic Viewpoint: Towards Formalization of the Semantics of Generics. Journal of Psycholinguistic Research, 51(6), 1209–1229. 10.1007/s10936-022-09851-1 35988112 PMC9646602

[80] Schulze, C., & Hertwig, R. (2021). A description–experience gap in statistical intuitions: Of smart babies, risk-savvy chimps, intuitive statisticians, and stupid grown-ups. Cognition, 210, 104580. 10.1016/j.cognition.2020.104580 33667974

[81] Silva, P. (2020). A Bayesian explanation of the irrationality of sexist and racist beliefs involving generic content. Synthese, 197(6), 2465–2487. 10.1007/s11229-018-1813-9

[82] Simms, L. J., Zelazny, K., Williams, T. F., & Bernstein, L. (2019). Does the number of response options matter? Psychometric perspectives using personality questionnaire data. Psychological Assessment, 31(4), 557–566. 10.1037/pas0000648 30869956

[83] Slovic, P., Finucane, M. L., Peters, E., & MacGregor, D. G. (2004). Risk as Analysis and Risk as Feelings: Some Thoughts about Affect, Reason, Risk, and Rationality. Risk Analysis, 24(2), 311–322. 10.1111/j.0272-4332.2004.00433.x 15078302

[84] Sterken, R. K. (2015). Generics in Context. Philosopher’s Imprint, 15(21). http://hdl.handle.net/2027/spo.3521354.0015.021

[85] Tasimi, A., Gelman, S. A., Cimpian, A., & Knobe, J. (2017). Differences in the Evaluation of Generic Statements About Human and Non-Human Categories. Cognitive Science, 41(7), 1934–1957. 10.1111/cogs.12440 27886394

[86] Tessler, M. H., & Goodman, N. D. (2019a). Learning from Generic Language. PsyArXiv. 10.31234/osf.io/hnm8p 42658583

[87] Tessler, M. H., & Goodman, N. D. (2019b). The language of generalization. Psychological Review, 126(3), 395–436. 10.1037/rev0000142 30762385

[88] Van Rooij, R., & Schulz, K. (2020). Generics and typicality: A bounded rationality approach. Linguistics and Philosophy, 43(1), 83–117. 10.1007/s10988-019-09265-8

[89] Verbree, A.-R., Toepoel, V., & Perada, D. (2020). The effect of seriousness and device use on data quality. Social Science Computer Review, 38(6), 720–738. 10.1177/0894439319841027

[90] Wickham, H. (2016). Ggplot2: Elegant graphics for data analysis (2nd ed.) [PDF]. Springer International Publishing.

[91] Wickham, H., Averick, M., Bryan, J., Chang, W., D’Agostino McGowan, L., François, R., Grolemund, G., et al. (2019). Welcome to the Tidyverse. Journal of Open Source Software, 4(43), 1686. 10.21105/joss.01686

[92] Wickham, H., & Bryan, J. (2025). readxl: Read Excel Files (Version 1.4.5). https://cran.r-project.org/web/packages/readxl/index.html

[93] Wickham, H., Hester, J., Francois, R., Bryan, J., & Bearrows, S. (2025). readr: Read Rectangular Text Data (Version 2.1.6). https://cran.r-project.org/web/packages/readr/index.html

[94] Xu, F., & Garcia, V. (2008). Intuitive statistics by 8-month-old infants. Proceedings of the National Academy of Sciences, 105(13), 5012–5015. 10.1073/pnas.0704450105 PMC2278207 18378901

[95] Zadeh, L. A. (1983). A computational approach to fuzzy quantifiers in natural languages. Computers & Mathematics with Applications, 9(1), 149–184. 10.1016/0898-1221(83)90013-5

