# Supplementary material for: When Do Generics Feel Justifiable? A Registered Report Bridging Key Theories
Source: J Cogn. 2026 Mar 5;9(1):21. doi: 10.5334/joc.493 (PMC12962254; doi:10.5334/joc.493)
Supplement: Supplementary Materials. — Supplemental file including the feature list in English (with Dutch translations), the alien name list, and an overview of the full results for both the pre-registered and explorative analyses. [file joc-9-1-493-s1.pdf]

Supplementary Materials

Feature list in English, with Dutch translations (from Cella et al., 2022, Experiment 3a):

<u>Non-dangerous features</u>

1. Sleep under trees *(slapen onder bomen)*
2. Stamp their feet to greet others *(stampen hun voeten om anderen te begroeten)*
3. Leave their leftovers on the ground *(laten hun restjes op de grond liggen)*
4. Run in zigzag patterns *(lopen in zigzag patronen)*
5. Swim when it’s raining *(zwemmen wanneer het regent)*
6. Are short *(zijn klein)*
7. Are messy *(zijn rommelig)*
8. Are skillful *(zijn handig)*
9. Are hairy *(zijn behaard)*

<u>Dangerous features</u>

1. Kill for fun *(doden voor plezier)*
2. Attack people *(vallen mensen aan)*
3. Kidnap babies *(ontvoeren jongen)*
4. Assault for no reason *(vallen zonder reden aan)*
5. Hunt strangers *(jagen op onbekenden)*
6. Are brutal *(zijn wreed)*
7. Are ruthless *(zijn genadeloos)*
8. Are bloodthirsty *(zijn bloeddorstig)*
9. Are violent *(zijn gewelddadig)*

List of alien names (collected from a number of sources):

1) Ackeps; 2) Aneds; 3) Bemes; 4) Blins; 5) Bripets; 6) Eders; 7) Elleps; 8) Feps; 9) Frams; 10) Gezos; 11) Glippets; 12) Heabs; 13) Javs; 14) Kazzes; 15) Kerns; 16) Lilvirs; 17) Lorches; 18) Ludins; 19) Moxes; 20) Nares; 21) Ollers; 22) Pilaks; 23) Stades; 24) Stups; 25) Tarbs; 26) Tembas; 27) Thups; 28) Toabos; 29) Trufts; 30) Upos; 31) Whezas; 32) Wuptos; 33) Yoxas; 34) Zavs; 35) Zorbs; 36) Zoovs

Summary of Results from Pre-registered Analysis Plan

Demographics before any exclusions

- ☐ Sample size
  - Sample size: *N* = 244
- ☐ Descriptive statistics for gender and age
  - Gender: 36 men, 207 women, 1 X
  - Age: *M* = 18.49, SD = 1.07, range = 17-25

Exclusions based on outcome neutral tests

1. Distribution of responses to practice trial
   - ☐ 64 participants responded below the midpoint of the scale (much more than expected – perhaps also do analyses with these participants included (exploratively)
2. Floor & ceiling effects
   - ☐ No participants affected by this criterion
3. Reaction times
   - ☐ 6 participants too fast or too slow
4. Seriousness question
   - ☐ 5 participants responded ‘not at all seriously’

Pre-registered to report number of participants affected by each and to only conduct the analyses below if percentage of excluded participants is below 40%.

- ▪ Important: overlap possible between criteria
- ▪ In total 69 participants were identified for exclusion based on these pre-registered criteria (28.28%).

Demographics, after exclusions based on outcome neutral tests

- ☐ Sample size
  - Sample size: *N* = 175
- ☐ Descriptive statistics for gender and age

- Gender: 24 men, 150 women, 1 X
- Age: *M* = 18.45, SD = 1.09, range = 17-25

Manipulation checks

- Absolute prevalence manipulation check
  - BS ANOVA: $F(2, 172) = 31.35$, $p < .001$, $\eta_p^2 = .267$
  - Follow-up contrast (Bonferroni corr. Welch's *t*-tests, with Cohen's d for independent samples)
    - Low vs Intermediate: $t(93) = -3.96$, $p < .001$, $d = -0.74$
    - Low vs High: $t(109) = -7.12$, $p < .001$, $d = -1.32$
    - Intermediate vs High: t(105) = -4.54, p < .001, $d = -0.84$
      - Low (n = 58): $M = 2.55$, $SD = 0.99$
      - Intermediate (n = 59): $M = 3.15$, $SD = 0.60$
      - High (n = 58): $M = 3.74$, $SD = 0.81$
- Relative prevalence manipulation check
  - BS ANOVA: $F(2, 172) = 7.29$, $p = .001$, $\eta_p^2 = .078$
  - Follow-up contrast (Bonferroni corr. Welch's *t*-tests, with Cohen's d for independent samples)
    - Negative vs Equal: $t(71.3) = -1.04$, $p = .909$, $d = -0.19$
    - Negative vs Positive: $t(118) = -3.11$, $p = .007$, $d = -0.57$
    - Equal vs Positive: t(69) = -3.16, p = .007, $d = -0.58$
      - Negative (n = 61): $M = -0.20$, $SD = 0.89$
      - Equal (n = 55): $M = -0.07$, $SD = 0.26$
      - Positive (n = 59): $M = 0.31$, $SD = 0.88$
- Dangerousness manipulation check
  - WS ANOVA: $F(1, 174) = 5033.83$, $p < .001$, $\eta_p^2 = .967$
  - Follow-up contrast (paired samples *t*-tests, with Cohen's d for dependent samples)
    - Non-dangerous vs Dangerous: $t(174) = -70.95$, $p < .001$, $d = -5.36$

- Non-dangerous: *M* = 0.67, *SD* = 0.43
- Dangerous: *M* = 3.41, *SD* = 0.37

Hypothesis tests

- 3x3x2 RM ANOVA
  - **Absolute prevalence**: $F(1.73, 300.17) = 1057.16$, $p < .001$, $\eta_p^2 = .859$
    - Follow-up contrast (Bonferroni corr. paired samples *t*-tests)
      - Low vs Intermediate: $t(174) = -21.90$, $p < .001$, $d = -1.66$
      - Low vs High: $t(174) = -38.73$, $p < .001$, $d = -2.93$
      - Intermediate vs High: $t(174) = -29.24$, $p < .001$, $d = -2.21$
        - Low: *M* = -1.31, *SD* = 0.98
        - Intermediate: *M* = -0.16, *SD* = 0.98
        - High: *M* = 1.40, *SD* = 0.83
  - **Relative prevalence**: $F(1.90, 329.74) = 22.54$, $p < .001$, $\eta_p^2 = .115$
    - Follow-up contrast (Bonferroni corr. paired samples *t*-tests)
      - Negative vs Equal: $t(174) = -3.42$, $p = .002$, $d = -0.26$
      - Negative vs Positive: $t(174) = -6.11$, $p < .001$, $d = -0.46$
      - Equal vs Positive: $t(174) = -3.65$, $p = .001$, $d = -0.28$
        - Negative: *M* = -0.15, *SD* = 1.45
        - Equal: *M* = -0.03, *SD* = 1.45
        - Positive: *M* = 0.11, *SD* = 1.45
  - Dangerousness: $F(1, 174) = 3.25$, $p = .073$, $\eta_p^2 = .018$
    - Follow-up contrast (paired samples *t*-test)
      - Non-dangerous vs Dangerous: $t(174) = -1.80$, $p = 0.073$, $d = -0.14$
        - Non-dangerous: *M* = -0.051, *SD* = 1.45
        - Dangerous: *M* = 0.001, *SD* = 1.46
  - Absolute prevalence * Relative prevalence: $F(3.79, 658.82) = 1.42$, $p = .229$, $\eta_p^2 = .008$

- **Absolute prevalence * Dangerousness**: F(1.78, 310.52) = 3.31, p = .043 , η_p^2 = .019
  - Follow-up contrasts (Bonferroni corr. paired samples t-tests, non-dangerous versus dangerous for different absolute prev. levels)
    - For AP = Low: t(174) = -0.53, p = 1.00, d = -0.04
      - Low x Non-dangerous: M = -1.33, SD = 0.96
      - Low x Dangerous: M = -1.30, SD = 1.01
    - For AP = Intermediate: t(174) = -2.75, p = 0.020, d = -0.21
      - Intermediate x Non-dangerous: M = -0.23, SD = 0.97
      - Intermediate x Dangerous: M = -0.09, SD = 0.98
    - For AP = High: t(174) = 0.48, p = 1.00, d = 0.04
      - High x Non-dangerous: M = 1.41, SD = 0.79
      - High x Dangerous: M = 1.39, SD = 0.87
- Relative prevalence * Dangerousness: F(2, 348) = 1.29, p = .28, η_p^2 = .007
- Absolute prevalence * Relative prevalence * Dangerousness: F(3.79, 659.80) = 1.60, p = .18, η_p^2 = .009

Check of humanness as a covariate

- Descriptives for humanness ratings: M = -0.38, SD = 1.38, Range: [-3,3]
- 3x3x2 RM ANCOVA (with humanness as a BS covariate)
  - Humanness: F(1, 173) = 0.31, p = .58, η_p^2 = .002
  - **Absolute prevalence**: F(1.72, 297.62) = 1059.33, p < .001, η_p^2 = .860
  - **Relative prevalence**: F(1.89, 327.68) = 22.43, p < .001, η_p^2 = .115
  - Dangerousness: F(1, 173) = 3.23, p = .074, η_p^2 = .018
  - Humanness * Absolute prevalence: F(1.72, 297.62) = 1.36, p = .26, η_p^2 = .008
  - Humanness * Relative prevalence: F(1.89, 327.68) = 0.19, p = .81, η_p^2 = .001
  - Humanness * Dangerousness: F(1, 173) = 0.0001, p = .99, η_p^2 = 7.69e-07
  - Absolute prevalence * Relative prevalence: F(3.78, 654.44) = 1.42, p = .23, η_p^2 = .008

- **Absolute prevalence * Dangerousness**: F(1.79, 308.95) = 3.30, p = . 044, η_p^2 = .019
- Relative prevalence * Dangerousness: F(2, 346) = 1.29, p = .28, η_p^2 = .007
- Humanness * Absolute prevalence * Relative prevalence: F(3.78, 654.44) = 0.93, p = .44, η_p^2 = .005
- Humanness * Absolute prevalence * Dangerousness: F(1.79, 308.95) = 0.37, p = .67, η_p^2 = .002
- Humanness * Relative prevalence * Dangerousness: F(2, 346) = 0.50, p = .61, η_p^2 = .003
- Absolute prevalence * Relative prevalence * Dangerousness: F(3.79, 655.98) = 1.60, p = .18, η_p^2 =.009
- Humanness * Absolute prevalence * Relative prevalence * Dangerousness: F(3.79, 655.98) = 0.49, p = .73, η_p^2 = .003

Model comparisons (for all features)

- ☐ 2 plots: bars & histograms
- ☐ Fit statistics:
  - **Model 1: P(f|G)**
    - R-squared = 0.6614
    - RMSE = 0.80
    - MAE = 0.62
  - **Model 2: P(f|G) - P(f|¬G)**
    - R-squared = 0.0061
    - RMSE = 1.63
    - MAE = 1.33
  - **Model 3: P(f|G) / P(f)**
    - R-squared = 0.0060
    - RMSE = 1.75
    - MAE = 1.39

- **Model 4: P(f|G) / P(f|¬G)**
  - R-squared = 0.0556
  - RMSE = 1.87
  - MAE = 1.46
- **Model 5: (P(f|G) - P(f|¬G)) / (1 - P(f|¬G))**
  - R-squared = 0.0660
  - RMSE = 1.89
  - MAE = 1.47

Kappa's (inter-rater reliability) for open response suspicion checks rated by two independent raters

- ☐ Absolute prevalence: κ = .22
  - Interpretation according to Landis & Koch (1977): "Fair"
- ☐ Relative prevalence: κ = .76
  - Interpretation according to Landis & Koch (1977): "Substantial"
- ☐ Dangerousness: κ = .54
  - Interpretation according to Landis & Koch (1977): "Moderate"

Inconsistencies between the first two independent raters were solved by a third independent rater.

Demographics, after further exclusions based on suspicion checks (open responses)

- ☐ Sample size
  - Sample size: N = 147
- ☐ Descriptive statistics for gender and age
  - Gender: 20 men, 127 women
  - Age: M = 18.47, SD = 1.14, range = 17-25

Manipulation checks after further exclusions based on suspicion checks (open responses)

- ☐ Absolute prevalence manipulation check
  - BS ANOVA: $F(2, 144) = 29.03$, $p < .001$, $\eta_p^2 = .287$
  - Follow-up contrast (Bonferroni corr. Welch's t-tests, with Cohen's d for independent samples)

- Low vs Intermediate: *t*(81.07) = -4.13, *p* < .001, *d* = -0.83
- Low vs High: *t*(92.65) = -6.90, *p* < .001, *d* = -1.40
- Intermediate vs High: t(85.20) = -3.98, p < .001, *d* = -0.81
  - Low (n = 49): *M* = 2.51, *SD* = 0.97
  - Intermediate (n = 51): *M* = 3.18, *SD* = 0.62
  - High (n = 47): *M* = 3.77, *SD* = 0.82

- Relative prevalence manipulation check
  - BS ANOVA: *F*(2, 144) = 7.85, *p* = .001, η_p^2 = .098
  - Follow-up contrast (Bonferroni corr. Welch's *t*-tests, with Cohen's d for independent samples)
    - Negative vs Equal: *t*(58.31) = -1.06, *p* = .879, d = -0.21
    - Negative vs Positive: *t*(97.76) = -3.21, *p* = .005, d = -0.64

      Equal vs Positive: t(61.34) = -3.27, p = .005, d = -0.64
      - Negative (n = 49): *M* = -0.22, *SD* = 0.87
      - Equal (n = 47): *M* = -0.09, *SD* = 0.28
      - Positive (n = 51): *M* = 0.33, *SD* = 0.86
- Dangerousness manipulation check
  - WS ANOVA: *F*(1, 146) = 4349.66, *p* < .001, η_p^2 = .968
  - Follow-up contrast (paired samples *t*-tests, with Cohen's d for dependent samples)
    - Non-dangerous vs Dangerous: *t*(146) = -65.95, *p* < .001, *d* = -5.44
      - Non-dangerous: *M* = 0.70, *SD* = 0.42
      - Dangerous: *M* = 3.40, *SD* = 0.38

Hypothesis tests after further exclusions based on suspicion checks (open responses)

- 3x3x2 RM ANOVA
  - **Absolute prevalence**: *F*(1.73, 251.92) = 842.62, *p* < .001 , η_p^2 = .852
    - Follow-up contrast (Bonferroni corr. paired samples *t*-tests)
      - Low vs Intermediate: *t*(146) = -20.17 , *p* < .001, *d* = -1.66

- Low vs High: *t*(146) = -34.62, $p < .001$, *d* = -2.86
- Intermediate vs High: t(146) = -25.54, p < .001, *d* = -2.11
  - Low: *M* = -1.34, *SD* = 0.97
  - Intermediate: *M* = -0.15, *SD* = 0.99
  - High: *M* = 1.37, *SD* = 0.87
- **Relative prevalence**: *F*(1.88, 274.22) = 18.15, $p < .001$, $\eta_p^2 = .111$
  - Follow-up contrast (Bonferroni corr. paired samples *t*-tests)
    - Negative vs Equal: *t*(146) = -3.14, *p* = .006, *d* = -0.26
    - Negative vs Positive: *t*(146) = -5.52, $p < .001$, *d* = -0.46
    - Equal vs Positive: *t*(146) = -3.19, *p* = .005, *d* = -0.26
      - Negative: *M* = -0.17, *SD* = 1.43
      - Equal: *M* = -0.05, *SD* = 1.45
      - Positive: *M* = 0.09, *SD* = 1.47
- Dangerousness: *F*(1, 146) = 0.920, *p* = .339, $\eta_p^2 = .006$
  - Follow-up contrast (paired samples *t*-test)
    - Non-dangerous vs Dangerous: *t*(146) = -0.96, *p* = 0.339, *d* = -0.08
      - Non-dangerous: *M* = -0.06, *SD* = 1.45
      - Dangerous: *M* = 0.03, *SD* = 1.46
- Absolute prevalence * Relative prevalence: *F*(4, 584) = 2.11, *p* = .79, $\eta_p^2 = .014$
- Absolute prevalence * Dangerousness: *F*(1.84, 268.60) = 3.08, *p* = .052 , $\eta_p^2 = .021$
  - Follow-up contrasts (Bonferroni corr. paired samples *t*-tests, non-dangerous versus dangerous for different absolute prev. levels)
    - For AP = Low: *t*(146) = -0.19, *p* = 1.00, *d* = -0.02
      - Low x Non-dangerous: *M* = -1.34, *SD* = 0.95
      - Low x Dangerous: *M* = -1.33, *SD* = 0.99
    - For AP = Intermediate: *t*(146) = -2.20, *p* = 0.088, *d* = -0.18
      - Intermediate x Non-dangerous: *M* = -0.21, *SD* = 0.97

        - Intermediate x Dangerous: *M* = -0.09, *SD* = 0.99
    - For AP = High: *t*(146) = 1.19, *p* = .714, *d* = 0.10
        - High x Non-dangerous: *M* = 1.39, *SD* = 0.83
        - High x Dangerous: *M* = 1.35, *SD* = 0.90
- Relative prevalence * Dangerousness: *F*(2, 292) = 1.08, *p* = .34, η_p^2 = .007
- Absolute prevalence * Relative prevalence * Dangerousness: *F*(4, 584) = 1.42, *p* = .23, η_p^2 = .010

Check of humanness as a covariate after further exclusions based on suspicion checks (open responses)

- Descriptives for humanness ratings: M = -0.42, SD = 1.39, Range: [-3,3]
- 3x3x2 RM ANCOVA (with humanness as a BS covariate)
    - Humanness: *F*(1, 145) = 1.17, *p* = .28, η_p^2 = .008
    - **Absolute prevalence**: *F*(1.72, 249.17) = 840.57, *p* < .001 , η_p^2 = .853
    - **Relative prevalence**: *F*(1.87, 271.85) = 18.09, *p* < .001, η_p^2 = .111
    - Dangerousness: *F*(1, 145) = 0.91, *p* = .341, η_p^2 = .006
    - Humanness * Absolute prevalence: *F*(1.72, 249.17) = 0.65, *p* = .50, η_p^2 = .004
    - Humanness * Relative prevalence: *F*(1.87, 271.85) = 0.56, *p* = .56, η_p^2 = .004
    - Humanness * Dangerousness: *F*(1, 145) = 0.003, *p* = .95, η_p^2 = 2.37e-05
    - Absolute prevalence * Relative prevalence: *F*(4, 580) = 2.11, *p* = .079, η_p^2 = .014
    - Absolute prevalence * Dangerousness: *F*(1.84, 266.87) = 3.06, *p* = .053, η_p^2 = .021
    - Relative prevalence * Dangerousness: *F*(2, 290) = 1.08, *p* = .34, η_p^2 = .007
    - Humanness * Absolute prevalence * Relative prevalence: *F*(4, 580) = 1.13, *p* = .34, η_p^2 = .008
    - Humanness * Absolute prevalence * Dangerousness: *F*(1.84, 266.87) = 0.20, *p* = .80, η_p^2 = .001

- Humanness * Relative prevalence * Dangerousness: $F(2, 290) = 0.76$, $p = .47$, $\eta_p^2 = .005$
- Absolute prevalence * Relative prevalence * Dangerousness: $F(4, 580) = 1.42$, $p = .23$, $\eta_p^2 = .010$
- Humanness * Absolute prevalence * Relative prevalence * Dangerousness: $F(4, 580) = 0.84$, $p = .53$, $\eta_p^2 = .006$

Model comparisons (for all features) after further exclusions based on suspicion checks (open responses)

- ☐ 2 plots: bars & histograms
- ☐ Fit statistics:
  - **Model 1: P(f|G)**
    - R-squared = 0.6528
    - RMSE = 0.81
    - MAE = 0.63
  - **Model 2: P(f|G) - P(f|¬G)**
    - R-squared = 0.0060
    - RMSE = 1.63
    - MAE = 1.33
  - **Model 3: P(f|G) / P(f)**
    - R-squared = 0.0066
    - RMSE = 1.76
    - MAE = 1.39
  - **Model 4: P(f|G) / P(f|¬G)**
    - R-squared = 0.0576
    - RMSE = 1.88
    - MAE = 1.46
  - **Model 5: (P(f|G) - P(f|¬G)) / (1 - P(f|¬G))**

- R-squared = 0.0617
- RMSE = 1.88
- MAE = 1.47

Summary of Explorative Results

Remove exclusion criterion for first outcome neutral test (explorative)

- ☐ Disregard exclusions for first outcome neutral test:
  1. Floor & ceiling effects
     - ☐ No participants affected by this criterion
  2. Reaction times
     - ☐ 6 participants too fast or too slow
  3. Seriousness question
     - ☐ 5 participants responded ‘not at all seriously’

Pre-registered to report number of participants affected by each and to only conduct the analyses below if percentage of excluded participants is below 40%.

- Important: overlap possible between criteria
- In total 11 participants identified for exclusion based on these explorative criteria (4.51 %).

Demographics, after exclusions based on stricter outcome neutral tests (explorative)

- ☐ Sample size
  - Sample size: N = 233
- ☐ Descriptive statistics for gender and age
  - Gender: 33 men, 199 women, 1 X
  - Age: M = 18.48, SD = 1.06, range = 17-25

Manipulation checks based on stricter outcome neutral tests (explorative)

- ☐ Absolute prevalence manipulation check
  - BS ANOVA: $F(2, 230) = 45.55$, $p < .001$, $\eta_p^2 = .284$

- Follow-up contrast (Bonferroni corr. Welch's *t*-tests, with Cohen's d for independent samples)
  - Low vs Intermediate: *t*(128.43) = -4.60, $p < .001$, *d* = -0.74
  - Low vs High: *t*(149.34) = -8.63, $p < .001$, *d* = -1.38
  - Intermediate vs High: t(141.62) = -5.61, p < .001, *d* = -0.90
    - Low (n = 78): *M* = 2.56, *SD* = 0.95
    - Intermediate (n = 77): *M* = 3.14, *SD* = 0.59
    - High (n = 78): *M* = 3.77, *SD* = 0.80

- Relative prevalence manipulation check
  - BS ANOVA: *F*(2, 230) = 6.41, $p = .002$, $\eta_p^2 = .053$
  - Follow-up contrast (Bonferroni corr. Welch's *t*-tests, with Cohen's d for independent samples)
    - Negative vs Equal: *t*(94.18) = -0.35, $p = 1.00$, *d* = -0.05
    - Negative vs Positive: *t*(152.60) = -2.73, $p = .021$, *d* = -0.44
    - Equal vs Positive: t(87.77) = -3.31, p = .004, *d* = -0.54
      - Negative (n =80): *M* = -0.1, *SD* = 0.87
      - Equal (n = 78): *M* = -0.08, *SD* = 0.27
      - Positive (n = 75): *M* = 0.27, *SD* = 0.86
- Dangerousness manipulation check
  - WS ANOVA: *F*(1, 232) = 5605.45, $p < .001$, $\eta_p^2 = .960$
  - Follow-up contrast (paired samples *t*-tests, with Cohen's d for dependent samples)
    - Non-dangerous vs Dangerous: *t*(232) = -74.87, $p < .001$, *d* = -4.90
      - Non-dangerous: *M* = 0.65, *SD* = 0.45
      - Dangerous: *M* = 3.39, *SD* = 0.36

Hypothesis tests based on stricter outcome neutral tests (explorative)

- 3x3x2 RM ANOVA
  - **Absolute prevalence**: *F*(1.73, 400.36) = 1307.69, $p < .001$, $\eta_p^2 = .849$

- Follow-up contrast (Bonferroni corr. paired samples *t*-tests)
  - ☐ Low vs Intermediate: $t(232) = -23.62$, $p < .001$, $d = -1.55$
  - ☐ Low vs High: $t(232) = -43.02$, $p < .001$, $d = -2.82$
  - ☐ Intermediate vs High: t(232) = -33.13, p < .001, $d = -2.17$
    - Low: $M = -1.32$, $SD = 0.99$
    - Intermediate: $M = -0.22$, $SD = 0.98$
    - High: $M = 1.35$, $SD = 0.87$
- **Relative prevalence**: $F(1.91, 442.97) = 27.65$, $p < .001$, $\eta_p^2 = .106$
  - Follow-up contrast (Bonferroni corr. paired samples *t*-tests)
    - ☐ Negative vs Equal: $t(232) = -3.52$, $p = .002$, d = -0.23
    - ☐ Negative vs Positive: $t(232) = -6.73$, $p < .001$, d = -0.44
    - ☐ Equal vs Positive: $t(232) = -4.32$, $p < .001$, d = -0.28
      - Negative: $M = -0.19$, $SD = 1.44$
      - Equal: $M = -0.07$, $SD = 1.44$
      - Positive: $M = 0.07$, $SD = 1.45$
- **Dangerousness**: $F(1, 232) = 5.08$, $p = .025$, $\eta_p^2 = .021$
  - Follow-up contrast (paired samples *t*-test)
    - ☐ Non-dangerous vs Dangerous: $t(232) = -2.25$, $p = .025$, $d = -0.15$
      - Non-dangerous: $M = -0.09$, $SD = 1.45$
      - Dangerous: $M = -0.04$, $SD = 1.45$
- Absolute prevalence * Relative prevalence: $F(3.78, 878.09) = 1.58$, $p = .18$, $\eta_p^2 = .007$
- **Absolute prevalence * Dangerousness**: $F(1.89, 438.46) = 3.70$, $p = .028$, $\eta_p^2 = .016$
  - Follow-up contrasts (Bonferroni corr. paired samples *t*-tests, non-dangerous versus dangerous for different absolute prev. levels)
    - ☐ For AP = Low: $t(232) = -1.33$, $p = 0.555$, $d = -0.09$
      - Low x Non-dangerous: $M = -1.35$, $SD = 0.96$
      - Low x Dangerous: $M = -1.29$, $SD = 1.03$

- ☐ For AP = Intermediate: *t*(232) = -2.85, *p* = .014, *d* = -0.19
  - Intermediate x Non-dangerous: *M* = -0.29, *SD* = 0.98
  - Intermediate x Dangerous: *M* = -0.16, *SD* = 0.99
- ☐ For AP = High: *t*(232) = 0.69, *p* = 1, *d* = 0.05
  - High x Non-dangerous: *M* = 1.36, *SD* = 0.83
  - High x Dangerous: *M* = 1.34, *SD* = 0.90

- Relative prevalence * Dangerousness: *F*(2, 464) = 1.97, *p* = .14, η_p^2 = .008
- Absolute prevalence * Relative prevalence * Dangerousness: *F*(3.83, 889.20) = 0.90, *p* = .46, η_p^2 = .004

Check of humanness as a covariate based on stricter outcome neutral tests (explorative)

- ☐ 3x3x2 RM ANCOVA (with humanness as a BS covariate)
  - Humanness: *F*(1, 231) = 0.75, *p* = .39, η_p^2 = .003
  - **Absolute prevalence**: *F*(1.71, 395.44) = 1312.85, *p* < .001, η_p^2 = .850
  - **Relative prevalence**: *F*(1.91, 440.88) = 27.55, *p* < .001, η_p^2 = .107
  - **Dangerousness**: *F*(1, 231) = 5.06, *p* = .025, η_p^2 = .021
  - Humanness * Absolute prevalence: *F*(1.71, 395.44) = 1.92, *p* = .16, η_p^2 = .008
  - Humanness * Relative prevalence: *F*(1.91, 440.88) = 0.21, *p* = .80, η_p^2 = .001
  - Humanness * Dangerousness: *F*(1, 231) = 0.14, *p* = .71, η_p^2 = .001
  - Absolute prevalence * Relative prevalence: *F*(3.78, 873.45) = 1.58, *p* = .18, η_p^2 = .007
  - **Absolute prevalence * Dangerousnes**s: *F*(1.89, 436.94) = 3.70, *p* = .028, η_p^2 = .016
  - Relative prevalence * Dangerousness: *F*(2.00, 462) = 1.97, *p* = .14, η_p^2 = .008
  - Humanness * Absolute prevalence * Relative prevalence: *F*(3.78, 873.45) = 0.98, *p* = .41, η_p^2 = .004
  - Humanness * Absolute prevalence * Dangerousness: *F*(1.89, 436.94) = 0.70, *p* = .49, η_p^2 = .003

- Humanness * Relative prevalence * Dangerousness: $F(2, 462) = 0.42$, $p = .66$, $\eta_p^2 = .002$
- Absolute prevalence * Relative prevalence * Dangerousness: $F(3.84, 886.22) = 0.90$, $p = .46$, $\eta_p^2 = .004$
- Humanness * Absolute prevalence * Relative prevalence * Dangerousness: $F(3.84, 886.22) = 0.74$, $p = .56$, $\eta_p^2 = .003$

Model comparisons based on stricter outcome neutral tests (explorative)

- ☐ 2 plots: bars & histograms
- ☐ Fit statistics:
  - **Model 1: P(f|G)**
    - R-squared = 0.6444
    - RMSE = 0.82
    - MAE = 0.63
  - **Model 2: P(f|G) - P(f|¬G)**
    - R-squared = 0.0057
    - RMSE = 1.62
    - MAE = 1.33
  - **Model 3: P(f|G) / P(f)**
    - R-squared = 0.0062
    - RMSE = 1.75
    - MAE = 1.39
  - **Model 4: P(f|G) / P(f|¬G)**
    - R-squared = 0.0549
    - RMSE = 1.86
    - MAE = 1.45
  - **Model 5: (P(f|G) - P(f|¬G)) / (1 - P(f|¬G))**
    - R-squared = 0.0641

- RMSE = 1.88
- MAE = 1.47
